# Propagation laws of discontinuous gas supply in the excavation roadway

**DOI:** 10.1371/journal.pone.0268453

**Published:** 2022-05-26

**Authors:** Ke Gao, Lianzeng Shi, Shengnan Li, Liangxiu Wen

**Affiliations:** 1 College of Safety Science and Engineering, Liaoning Technical University, Huludao, Liaoning, PR China; 2 Key Laboratory of Mine Thermodynamic Disasters and Control of Ministry of Education, Liaoning Technical University, Huludao, Liaoning, PR China; NUST: National University of Sciences and Technology, PAKISTAN

## Abstract

An explosion with a discontinuous gas supply (DGS-explosion) is more complicated than a common secondary explosion. We present the results of a study on the propagation laws of the DGS-explosion induced by a gas explosion in excavation roadways. A rectangular tube was established using ANSYS, similar to an excavation roadway in an underground coal mine. The gas, flame, and shock wave propagation laws were determined by analyzing the explosive gas as it exited the excavation roadway. The results show that the initial explosion caused the flame generated in the DGS-explosion to be significantly stretched. Moreover, the shock wave was reflected by the end of the tube, which resulted in the reverse migration of the local gas after the DGS-explosion. Meanwhile, with the increase in local gas concentrations, the pressure peak and the entire explosion system can increase after the DGS-explosion. The flame region, temperature peak, and flame irregularity in the tube positively correlate with the concentration. These results can provide theoretical support and an experimental basis for preventing and responding to accidents caused by gas explosion accidents.

## 1. Introduction

In China, gas explosion accidents are one of the most devastating accidents in coal mines. Although coal mine accidents in China have decreased significantly with the continuous improvement of mining technology, gas explosion accidents are still a significant part of coal mine production. As shown in Table 1, gas explosion accidents have occurred worldwide in recent years [1]. Although gas explosions’ prevention and control methods have been studied extensively [2], disasters still happen. A gas explosion may occur when the surrounding environment reaches ignition conditions [3, 4]. The high temperature and high pressure generated after a gas explosion may cause multiple explosions when the initial explosion comes into contact with methane gas due to poor ventilation management or local protrusion [5].

**Table 1:** Severe gas explosion in coal mines worldwide.

Country	Coal mine	Casualties	Date
USA	Upper Big Branch	29	04/05/2010
Russia	Raspadskaya	90	05/08/2010
Turkey	Karadon	30	05/17/2010
Colombia	San Fernando	73	06/16/2010
New Zealand	Pike River	29	11/19/2010
Pakistan	Dukki	52	03/20/2011
Ukraine	Sukhodilska-Skhidna	26	07/29/2011
China	Babao	53	03/29/2013
Ukraine	Soma Komur	301	07/29/2011
Ukraine	Zasyadko	33	03/04/2015
Russia	Severnaya	36	02/25/2016
China	Jinshangou	33	10/31/2016
China	Baoma	32	12/03/2016
Russia	Kemerovo	52	11/25/2021

In recent years, scientific knowledge of gas explosions and prevention measures have improved, both in China and other countries. There are many reviews and references to the laws of gas explosion propagation. The flame acceleration and microstructure, external conditions (such as obstacles [6, 7], gas concentration [8, 9], temperature [10, 11], and ignition energy [12, 13], etc.), as well as characteristic parameters during the gas explosion have been investigated. Shock waves comprise most of the energy, and compressing shock waves can cause an explosion of premixed gas. Many studies have been conducted on the impact of shock waves. Most gas explosions occur in the form of deflagration, and the flame generated by the detonating source forms a shock wave during the first propagation in the roadway. Shock waves are often accompanied by high temperature, explosive pressure, and speed, causing many casualties and severe destruction of the underground facilities.

**Fig 1:**
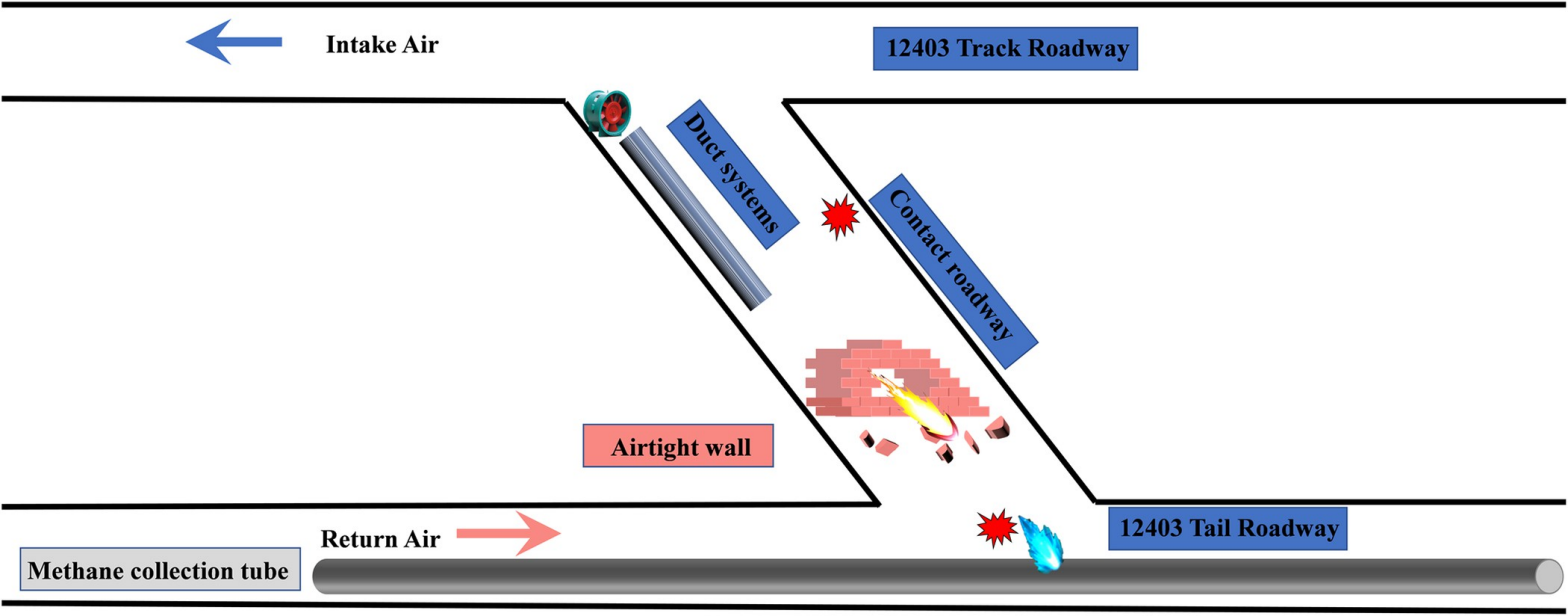
Schematic diagram of gas explosion location in Tunlan Coal Mine.

Coal mine explosions occur mainly at local gas accumulation locations. There is also the possibility of gas accumulation during shockwave propagation. The locally accumulated gas is ignited and explodes under the explosion shock wave and the high-temperature effect. The local flow field is then affected by the local gas explosion, which intensifies the intensity of the gas explosion shock wave and has the potential to expand the number of casualties and the degree of property loss. In 2019, a gas explosion occurred in the Tunlan Coal Mine of Xishan Coal and Electricity Group in Shanxi Province, China [14]. The initial explosion occurred in the 12403 track roadway and tail roadway (see Fig 1). The gas explosion energy damaged the drainage tube in a dedicated return-air roadway. Consequently, the high concentration and high equivalent gas in the tube added to the explosion, resulting in 87 deaths. This horrible accident was mainly caused by an explosion of gas inside the tube. Therefore, it is urgent to study explosion shock wave propagation under the condition of a local discontinuous gas supply along the route of gas explosion shock wave propagation. With a complete understanding of the characteristics and laws of gas explosions, improvements can be made in explosion disaster prevention and control.

In disasters involving multiple explosions, the shock wave is immediately formed after the initial explosion, which contains most of the energy released by the gas explosion. It can also cause the gas/air premixed gas to explode because of the compression of the shock wave [15]. Kiverin et al. demonstrated that one of the leading factors in the formation of multiple explosions is the state of non-steady flow dynamics behind the shock wave. After the initial ignition takes place, the formed reaction wave can ignite gas accumulated elsewhere. Shock-induced compression of a test mixture was shown to yield conditions for self-sustained acceleration and, ultimately, detonation [16]. Edyta and colleagues confirmed that the shock wave and boundary layer interactions are the keys to auto-ignition in the boundary layer in a smooth tube. In this situation, a new flame is generated, and the deflagration to detonation process (DDT) is induced [17]. Smirnov studied the detonation process caused by reflected waves inside a container through numerical simulations and experiments. The results showed that a detonation wave was formed in reflection and focusing and intermediate transient regimes [18–20]. Some researchers have analyzed the changes in state parameters of methane under shock wave compression and suggested that the higher the Mach number, the more pronounced the thermal decomposition of methane. And the overpressure of the deflagration wave increases with the increase of the shock wave Mach number in the release tube [21–23]. Zhang et al. [24] investigated the process of a complex two-dimensional flow field generated by the interaction when a plane shock wave entered an asymmetrical cavity with curved end walls and ignited the flame. Their experiment demonstrated that there are two types of ignition-weak and strong-in the container with the change in shock wave velocity.

In addition, there has been some research on the explosion of premixed gas induced by a high-temperature flame, where the interaction between the thermal ignition mechanism and the chain ignition mechanism can cause the explosion [25, 26]. High-temperature flames must meet two conditions simultaneously to induce a gas explosion [27]. One is that the flame temperature exceeds the minimum temperature of the explosive gas, and the other is that the flame duration exceeds the induction period of the gas explosion [28]. There are many experimental studies on gas explosions at different ignition sources. For example, it was reported in one study that the ignition source position had a significant influence on gas explosion [29]. Mohammed [30, 31] revealed the effect of different ignition energies on the gas explosion characteristics. Enis [32] determined the influence of strong ignition sources on explosion and decomposition. The results of these other studies point to the same conclusion: the higher the ignition energy intensity, the larger the heating area, and the longer the action time [33]. The wider the explosion limit range, the more mature the mechanism of flame-induced gas explosion [34]. Researchers [35, 36] have focused on the ignition method, ignition energy point explosion gas theory, and experimental research and have provided some theoretical and technical support for gas explosion shock waves and flame front coupling point explosions of local gas in the excavation roadway.

Taken together, the research to date leaves no doubt that a shock wave coupled with high temperature induces the explosion of the premixed gas. Considering all of these factors, let us explore the characteristics of gas explosions with a discontinuous gas supply (DGS). DGS-explosions refer to phenomena of multiple explosions in which a gas explosion source causes another explosion. The energy generated by the primary explosion affects the local flow field and enhances the intensity of the gas explosion shock wave. The scope of the DGS-explosion accident is thus further expanded, potentially causing many casualties and property losses. Notwithstanding the severity of these accidents, the relevant experimental data is scarce and relatively scattered. The mechanism behind DGS-explosions and the pertinent evaluation conditions have not received much attention. There is a lack of systematic research on the development law of DGS-explosions. Therefore, it is necessary to study the explosion caused by local gas ignited under the condition of DGS after the explosion has occurred in the heading face. The present study focused on the excavation roadway, and a numerical model was established. This numerical model determined the dynamic mechanical characteristics and changing laws of the shock wave and flame wave after a local explosion under DGS conditions. The results provide a theoretical basis for safety design in actual production.

## 2. Numerical parameters setting and experimental verification

### 2.1 Mathematical model and parameters

The gas explosion process is considered a fat-burning process in the tunnel, which follows the conservation laws of mass, energy, and chemical components. The gas explosion process uses a one-step reaction. The large eddy model (LES) was used to simulate the characteristics of turbulence [37, 38]. The laminar finite rate/eddy dissipation model in a turbulent premixed flame was used in the combustion process [39].

1. Mass conservation equation
  Because the mass change is neglected in the chemical reaction, the entire reaction system follows mass conservation, which can be written as
$$\frac{\partial \rho }{\partial t}+\frac{\partial (\rho v_{j})}{\partial x_{j}}=0$$ (1)
2. Momentum conservation equation
$$\rho \frac{dv_{j}}{dt}=-\frac{\partial P}{dx_{i}}+\frac{\partial }{dx_{j}}[\mu (\frac{\partial v_{j}}{\partial x_{i}}+\frac{\partial v_{i}}{\partial x_{k}})]-\frac{2}{3}\frac{\partial }{dx_{j}}[(\mu -\mu^{'})\frac{\partial v_{i}}{\partial x_{j}}]+\sum \rho_{s}F_{si}$$ (2)
3. Energy (enthalpy) conservation equation:
$$\rho C_{p}\frac{dT}{dt}-\frac{\partial P}{\partial t}=\frac{\partial }{\partial x_{i}}(\lambda \frac{\partial T}{\partial x_{j}})-\frac{\partial }{\partial x_{j}}[\sum_{s}\rho V_{sj}\int_{T_{0}}^{T}C_{ps}dT]+\frac{\partial q_{rj}}{\partial x_{j}}+\sum_{s}W_{s}h_{os}+\tau_{ij}\frac{\partial v_{i}}{\partial x_{j}}+\sum_{s}\rho_{s}F_{s}V_{s}$$ (3)
4. Mass fraction equation
$$\frac{\partial \rho \theta_{i}}{\partial t}+\frac{\partial }{\partial x_{j}}(\rho v_{j}\theta_{i}-\frac{\lambda_{t}}{\sigma_{e}}\frac{\partial \theta_{i}}{\partial x_{j}})=Hb_{T}Hb_{Fu}$$ (4)
  where *t* is time (s), *ρ* is density (kg·m^-3^), *v*_*i*_*、v*_*j*_ are mixed gas flow velocity (m·s^-1^); *P* is pressure (Pa), *μ* is dynamic viscosity (N·s·m^2^);∑*ρ*_*s*_*F*_*si*_ is the body force (*g*), *T* is the temperature (K), *λ* is the coefficient of viscosity (Pa·s), *W* is power (J), *τ*_*ij*_ is the viscous strain tensor (N·m^-1^); *h*_*os*_ is the enthalpy of formation (J·mol^-1^); *C*_*ps*_ is the specific heat (J·kg^-1^·K^-1^), *V*_*s*_ is the volume (m^3^), *θ*_*i*_ is mass percent concentration (%), Hb_T_ is a constant (1.1), and *σ*_*e*_ is the Prandtl number.
  To calculate turbulence, the K-ε model is used, in which it is assumed that the flow is completely turbulent, ignoring the influence of molecular viscosity. The two-equation K-ε model can be expressed as follows [40]:
$$\frac{\partial (\rho k)}{\partial t}+\frac{\partial (\rho ku_{i})}{\partial x_{i}^{}}=\frac{\partial }{\partial x_{j}}[(\mu +\frac{\mu_{t}}{\sigma_{k}})\frac{\partial k}{\partial x_{k}}]+G_{k}-\rho \varepsilon$$ (5)
$$\frac{\partial (\rho \varepsilon )}{\partial t}+\frac{\partial (\rho \varepsilon \mu_{i})}{\partial x_{i}}=\frac{\partial }{\partial x_{i}}[(\mu +\frac{\mu_{t}}{\sigma_{\varepsilon }})\frac{\partial \varepsilon }{\partial x_{j}}]+\frac{C_{1_{\varepsilon }}\varepsilon }{k}-C_{2\varepsilon }\rho \frac{\varepsilon^{2}}{k}$$ (6)
  where *k* is turbulent kinetic energy (J),*ε* is the turbulent dissipation rate, *μ*_*t*_ is the turbulent viscosity (Pa), and *G*_*k*_ is the generation item: σ_*k*_ = 1.0; σ_*ε*_ = 1.3; C_1ε_ = 1.44; C_2ε_ = 1.92.

### 2.2 Initial conditions and boundary conditions

In the present experiment, the initial concentration was 9.5vol.% methane-air premixed gas when the initial time was t_0_. The initial temperature of the entire flow field was 300 K, and the initial pressure was 0 Pa. The input parameters of the premixed gas are listed in Table 2. The non-slip insulation wall of the numerical model was adopted for the walls as boundary conditions. Ignition was realized by giving an initial temperature of 1,500 K.

**Table 2:** Initial parameter setting during numerical simulation.

Parameters	*ω*_CH4_	*ω*_O2_	*ω*_CO2_	*ω*_H2O_	P/MPa	T/K
Ignition area	0	0	0.145	0.118	1.01	1,500
Other areas	0.053	0.221	0.21	0	0	300

### 2.3 Numerical modeling verification by gas explosion tests

#### 2.3.1 Experimental apparatuses and procedure

**Fig 2:**
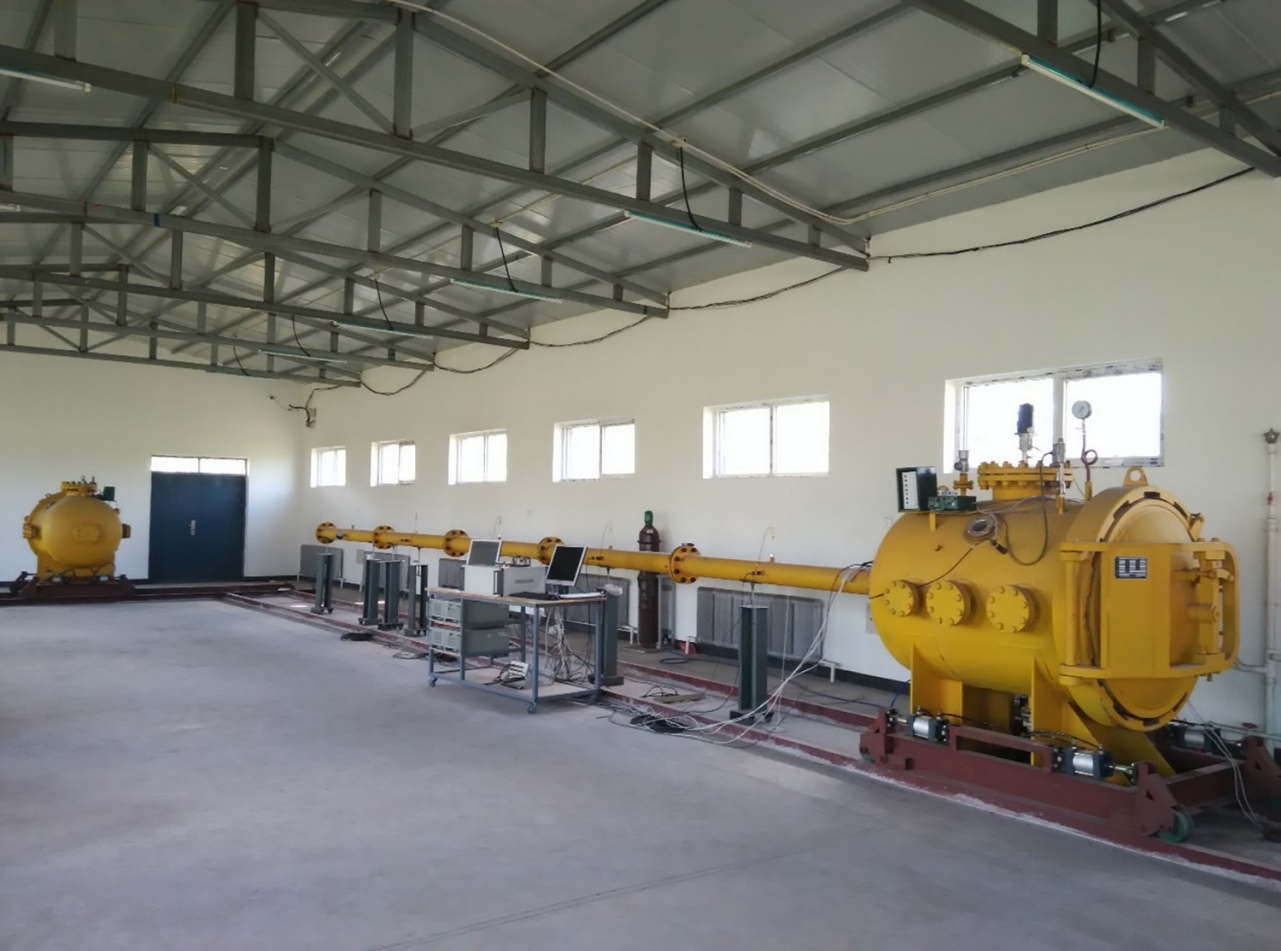
The testing platform of the tube gas explosion: Experimental setup.

**Fig 3:**
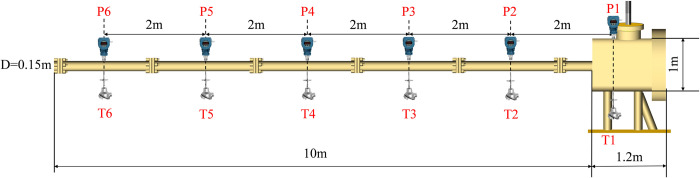
The testing platform of the tube gas explosion: Schematic diagram of experimental equipment.

The gas tube network explosion test platform of Liaoning Technical University consists of a device body, control system, and data acquisition system (Fig 2). The device’s body includes a 1-m^3^ volume of the anti-explosion tank and several anti-explosion tubes. The effective volume of the tank was 1 m^3^, the design pressure was 2.2 MPa, and the operating pressure was 2.0 MPa. The primary function of the control system is to trigger data sampling and safety chain monitoring. The core of the control system programmable logic controller (PLC) model for the Japanese Panasonic FPX-L14, which has 14 relay-type contacts data acquisition systems, mainly includes flame temperature data acquisition (the U.S. Nanmac production C2-7-K thermocouple) and explosion pressure data acquisition (U.S. Dytran piezoelectric high-sensitivity sensors). The monitoring location of pressure and temperature, the (M1-M6) point, is shown in Fig 3.

#### 2.3.2 Validation of numerical model with testing data

**Fig 4:**
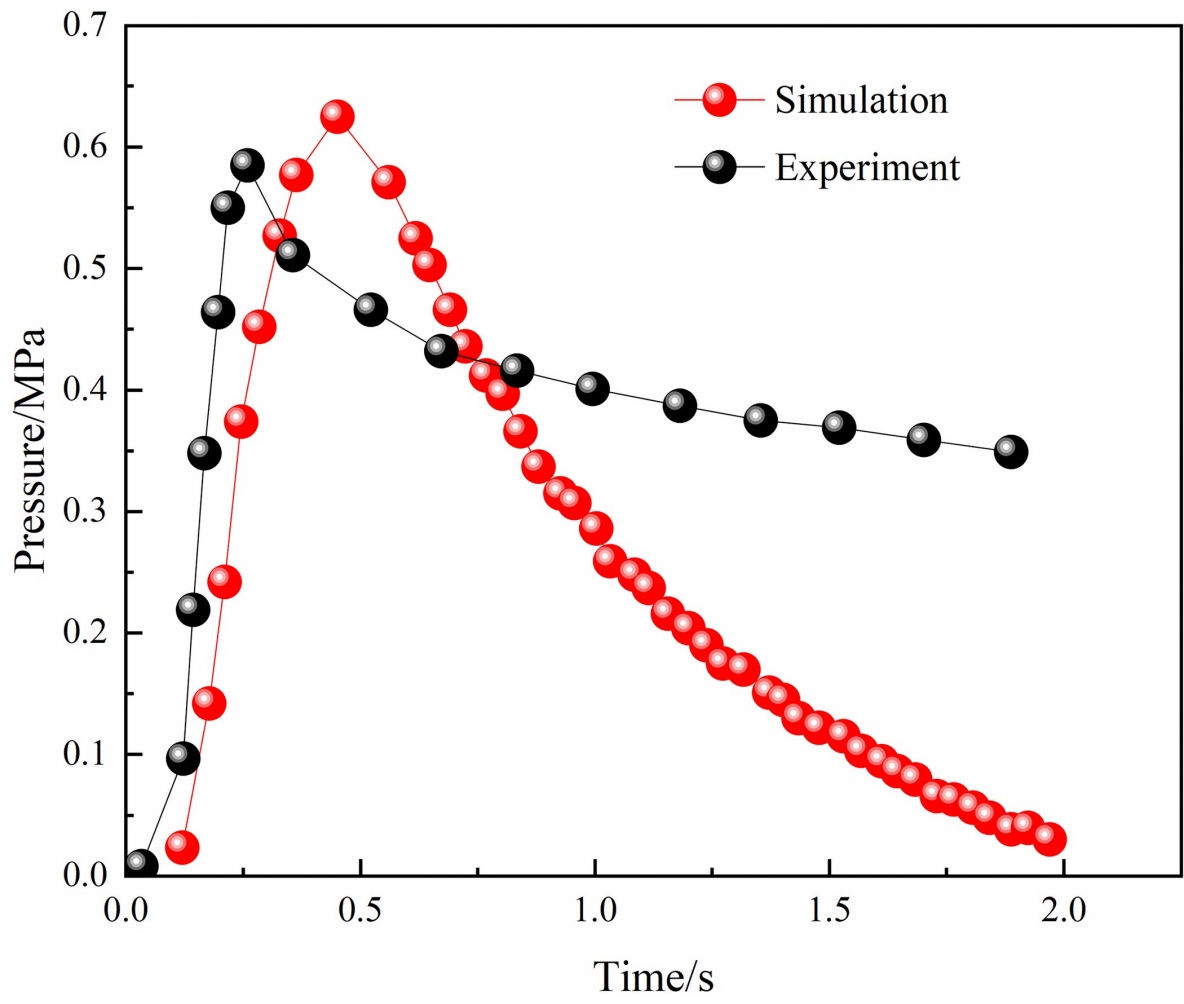
Experimental and simulant pressure comparison in M1.

**Fig 5:**
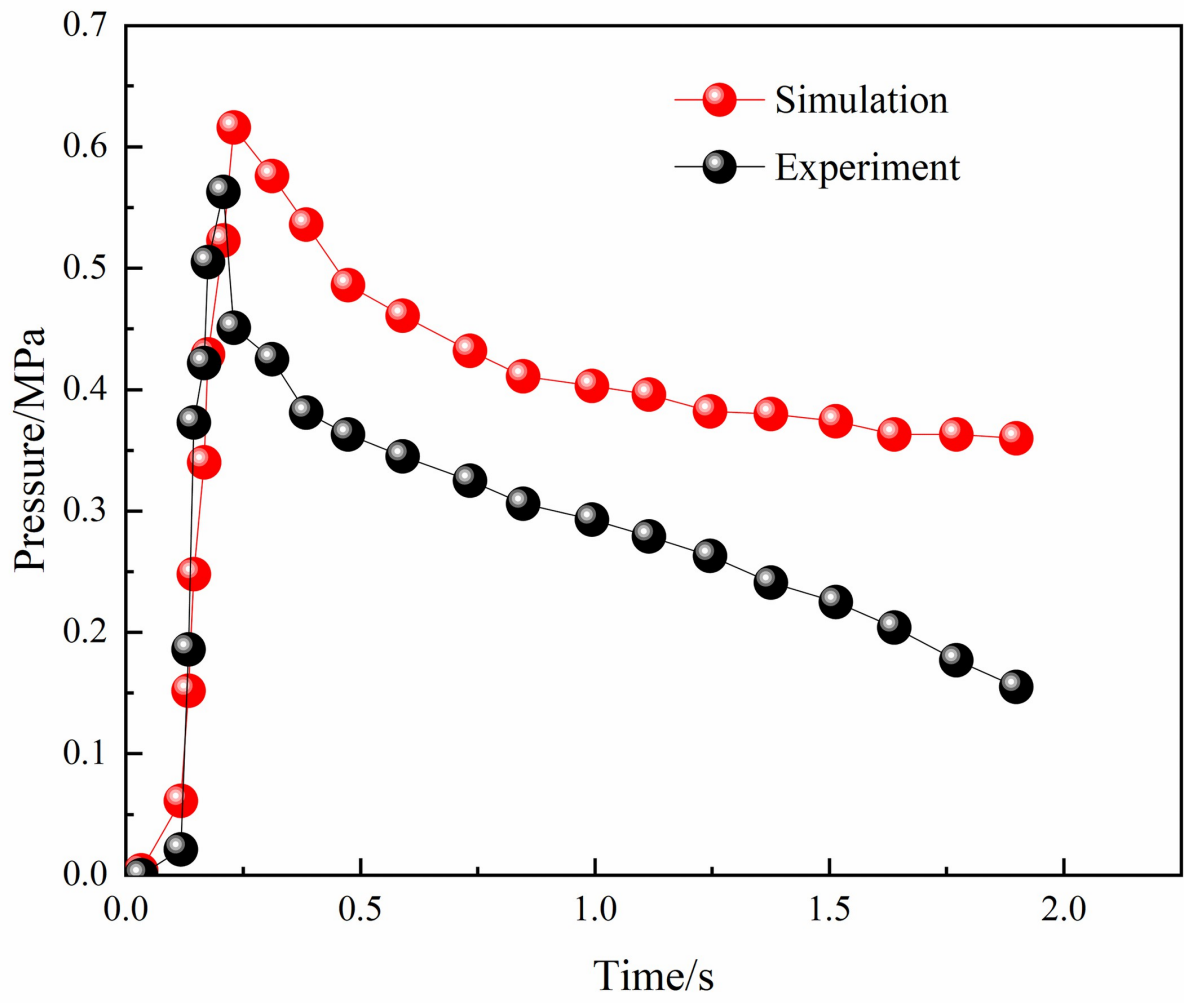
Experimental and simulant pressure comparison in M3.

**Fig 6:**
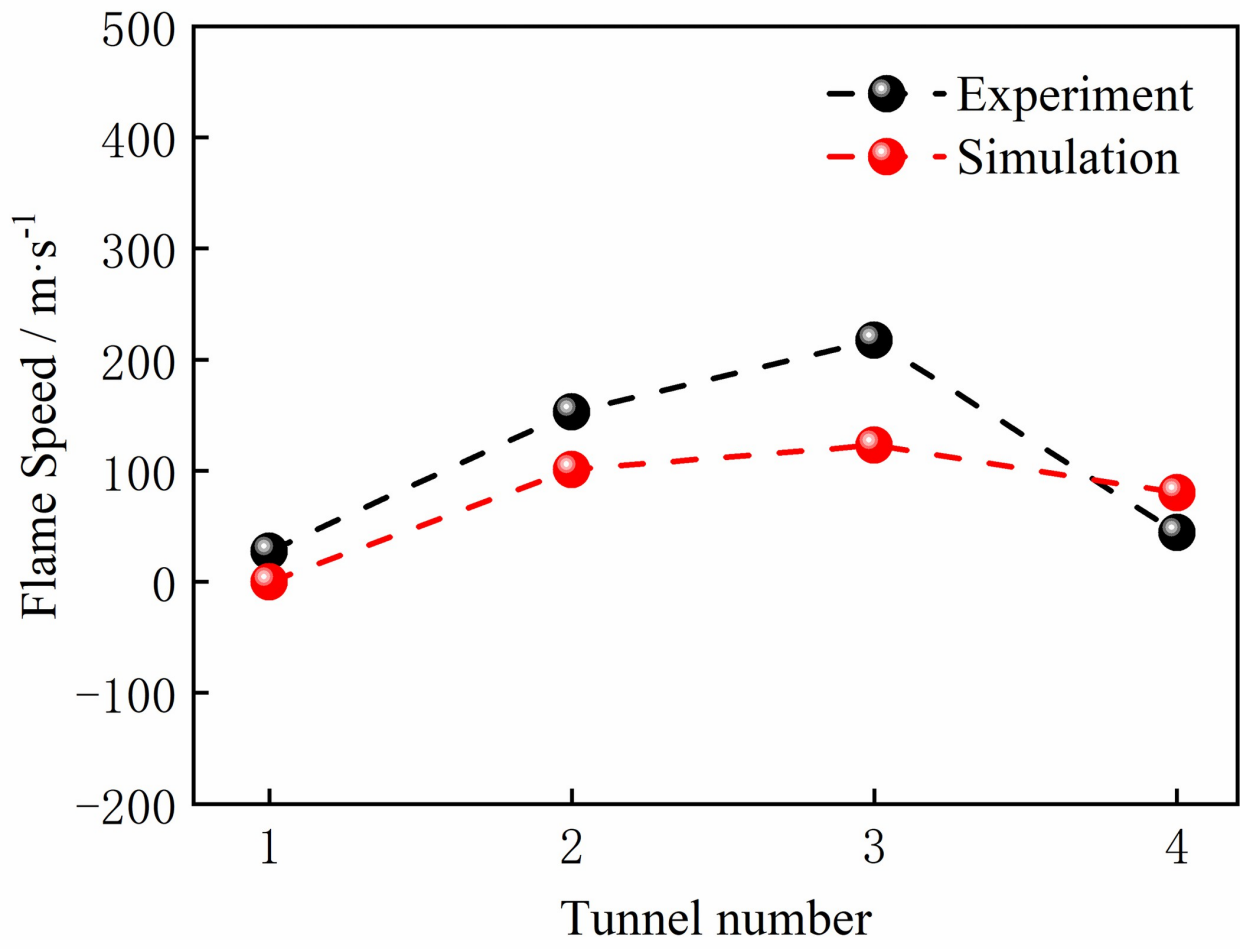
Experimental and simulant flame comparison at a concentration of 8.5vol.%.

**Fig 7:**
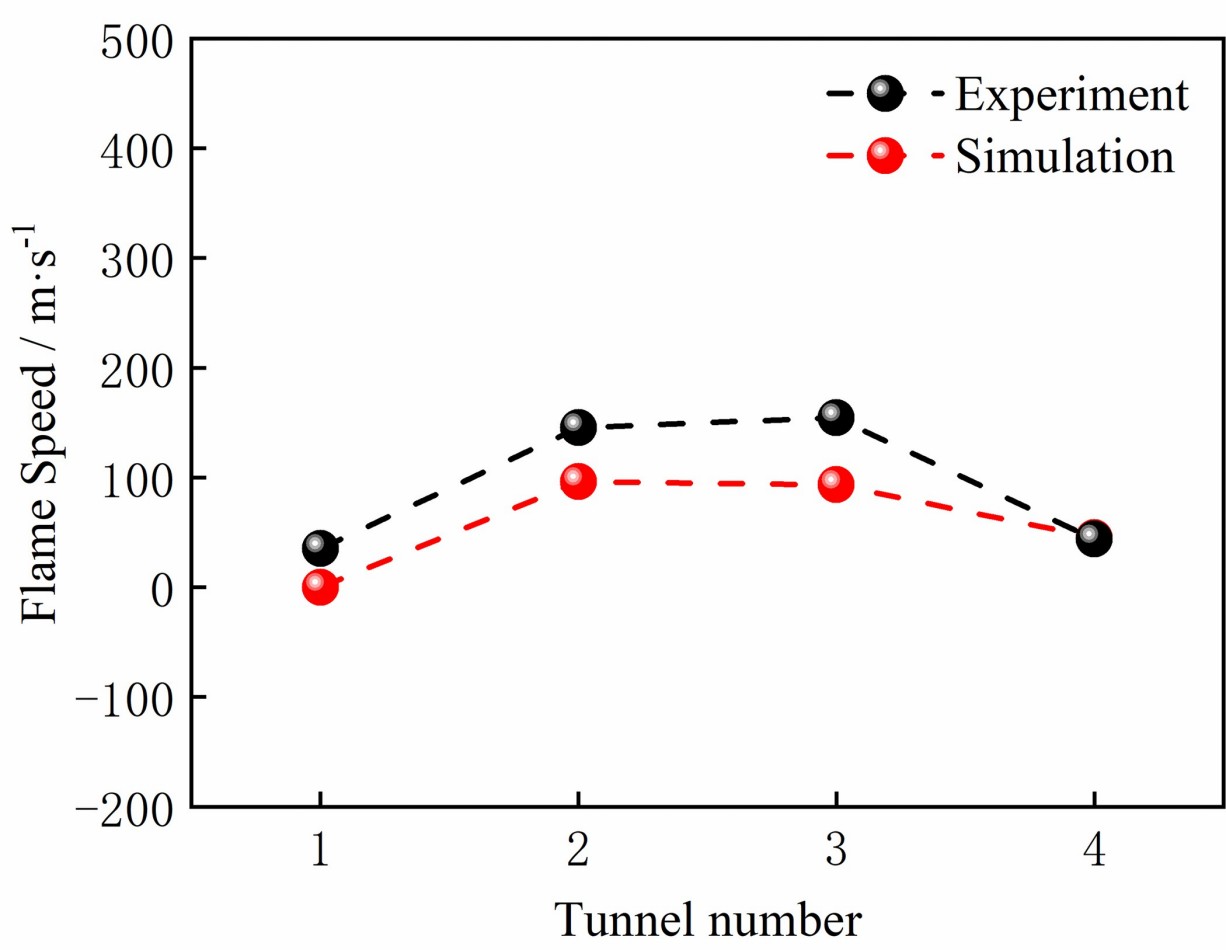
Experimental and simulant flame comparison at a concentration of 8.0vol.%.

We present in Figs 4 and 5 the simulated and experimental values of the explosion pressure at M1 and M3. These values generally agree with the trend, with a good agreement in the initial rise phase of the explosion pressure and a certain error in the decay phase. The minimum error between the experimental pressure peak at the monitoring point and the simulated pressure peak at monitoring point M3 was 2.5%. In addition, gas concentrations of 8.5% and 8% of the gas explosion flame speed experimental values and simulated values were compared. As shown in Figs 6 and 7, the flame propagates slowly in the reactor after ignition. The explosion flame then travels to the slender tube, where the flame propagation speed increases, but the flame propagation speed slows down in tube 4. Although there is a certain error between the flame propagation speed derived from the numerical simulation and the experimental physical value, the direction of the trend of these two variables was similar. A comprehensive analysis of the physical experiment and numerical simulation process showed the main reasons for the deviation. First, owing to the deviation of the readings and instruments, there was a certain error between the actual concentration in the model and the desired value during the experiment. In addition, although it was assumed that the gas explosion experiments took place in a confined space, the system valves and sealing caps may not have been tight enough, resulting in errors in the results. Second, the system wall was smooth and adiabatic in the numerical model. However, the actual tube wall is not smooth, and there was some heat dissipation, which might have led to deviations in the experimental results. Although there is some deviation between the numerical simulation and the physical experiments, the trend of the flame propagation speed was consistent. It can be considered that the numerical model selected in this study can effectively reflect the gas explosion propagation law.

#### 2.3.3 Grid independence

**Fig 8:**
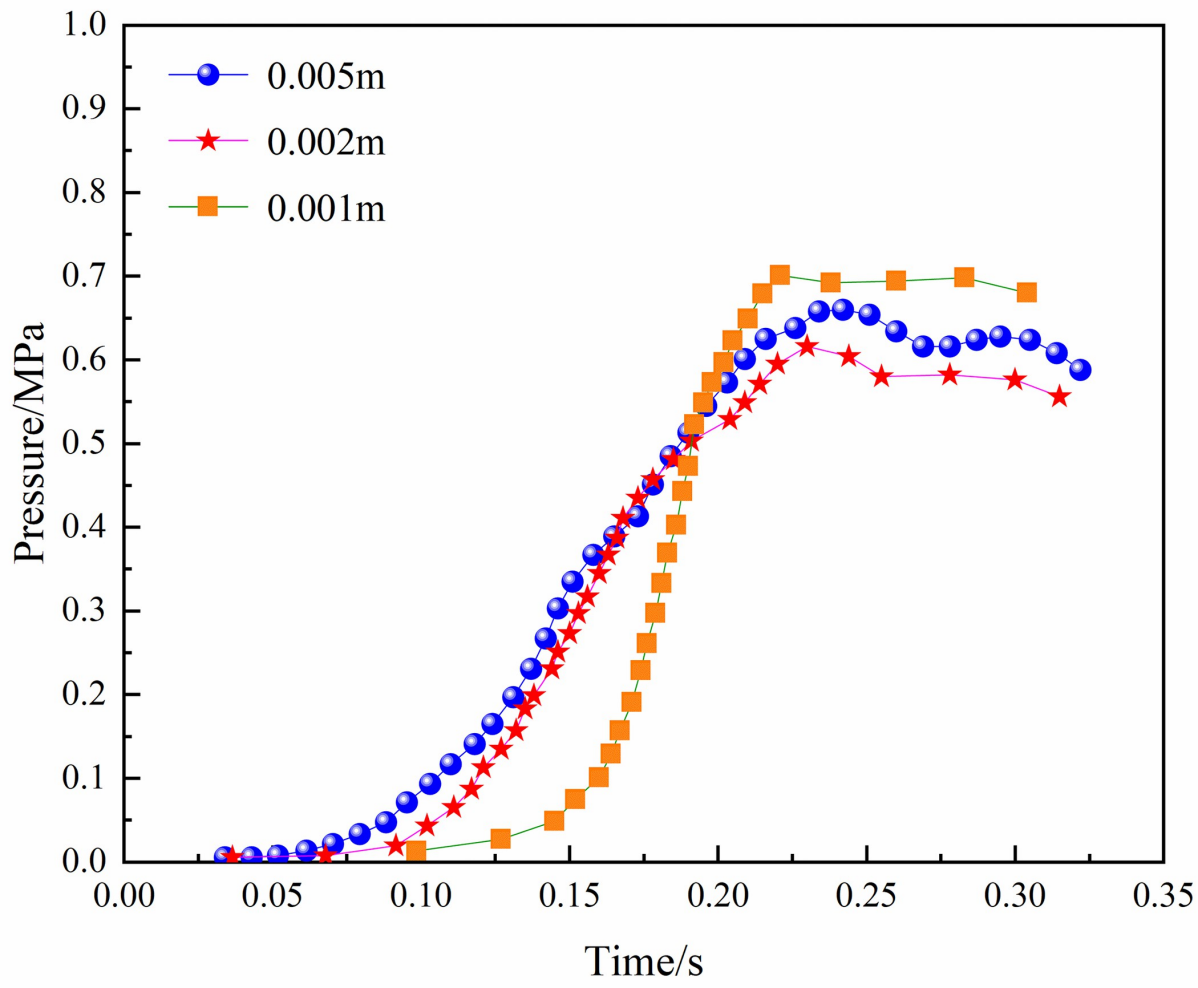
Pressure-time curves under different mesh size.

In large eddy simulations, the physical diffusion induced by the sub-grid model decreases as the length of the grid divided against the model is reduced. Therefore, the large eddy simulation requires a fine meshing of the model and the selection of a sufficiently smalltime step. The numerical spread of the large eddy simulation (LES) is much smaller than that of Reynolds Average Navier-Stokes (RANS). The actual model was simplified to obtain the simplified model shown below: The structured meshing was carried out for the above model, and the mesh sizes chosen were 0.005, 0.002, and 0.001 m. The variation in the gas explosion pressure in the model under different mesh sizes is shown in Fig 8. It can be seen from the figure that the trend in the explosion pressure curve is generally consistent between the 0.001 and 0.002 m grid sizes; the difference between the two is small. To reduce the calculation running time and improve the efficiency of the numerical calculation, a grid size of 0.002 m was chosen in this study, and the total number of grids divided by the model was 700,000.

## 3. Numerical simulation of the DGS-explosion

### 3.1 Physical modeling

**Fig 9:**
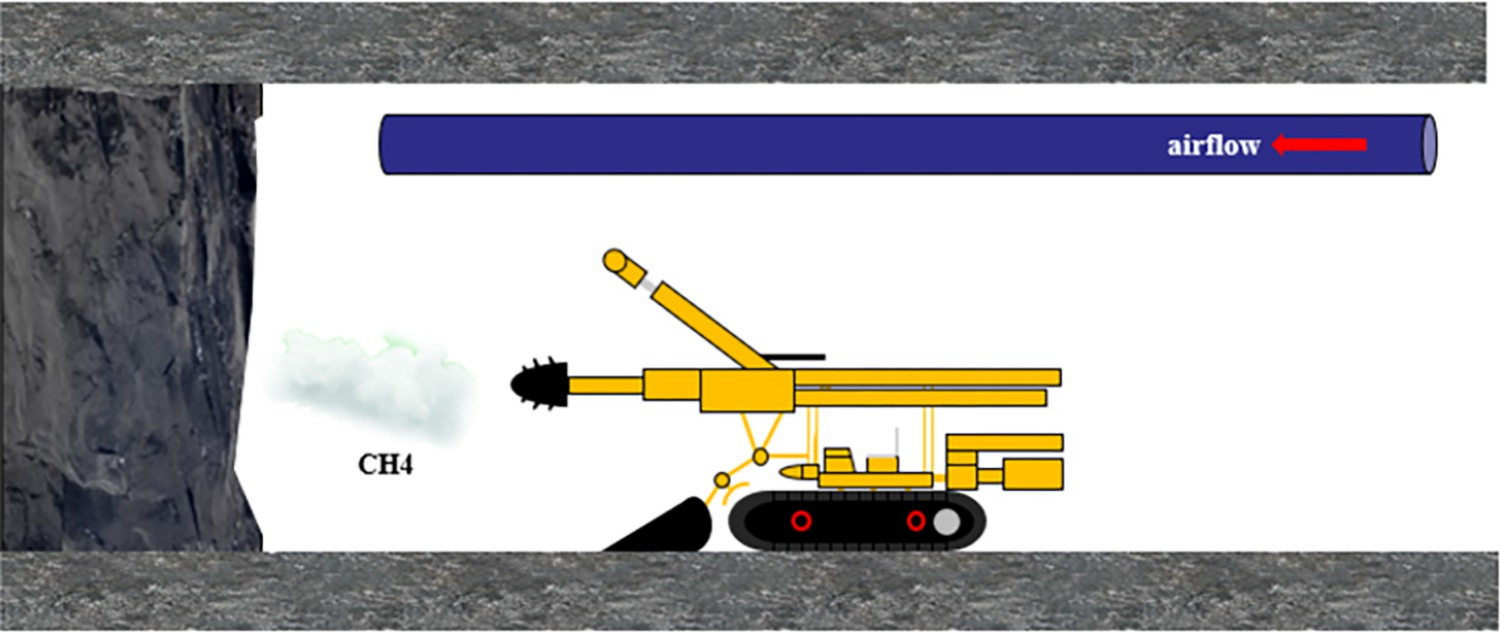
Engineering background.

**Fig 10:**
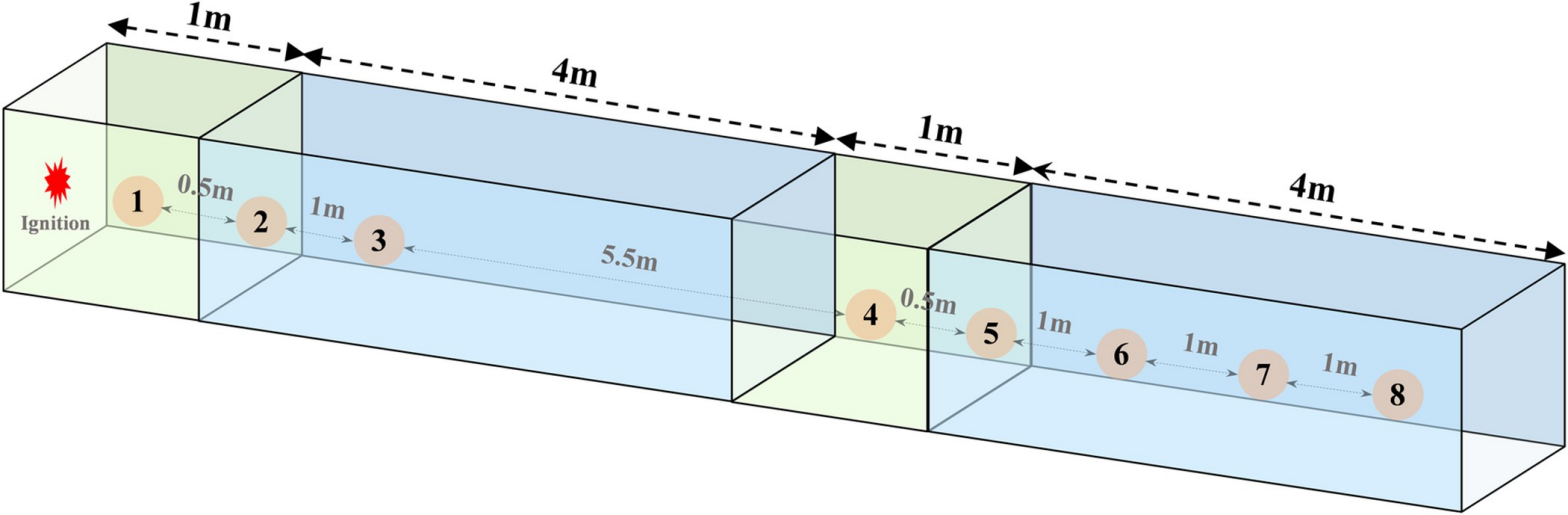
Explosion model in the tube.

As shown in Fig 9, the gas will gush out from the working face during the tunneling. After a gas explosion occurs at the working face, the replenished energy causes the local accumulation of gas to explode. Therefore, the excavation working face is considered as the study background, with which we can explore the energy characteristics of the explosion through experimental and numerical simulation methods. This study built a 3D rectangular model, a tube 10 m long and 1m in diameter. There are two separated gas zones in the model, one at the left end of the tube (initial gas) and the other near the center of the tube (local gas) (see Fig 10). The gas area is a cube with a length of 1m. The gas concentration at the end of the tube is set at 9.5%. The local gas concentration in the center of the tube changes with the working conditions, and the concentration range is 5% - 16%. The initial temperature of the simulation is 300 K, the grid unit is 0.002 m, and the ignition position is located at the center of the gas. A total of eight monitoring points were set; the monitoring point coordinates were (0.5, Y, 0.5), and the y values were 0.5, 1, 2, 5.5, 6, 7, 8, 9.

### 3.2 Meshing and simulation initial conditions

The size of the structural grid was 0.02 m, and the model consisted of 0.7 million grids. The tube wall was adiabatic and frictionless (the coefficient of thermal conductivity and friction coefficient were zero). The initial temperature and pressure were 300 K and 0 Pa, respectively. The initial speed of the entire area was 0 m·s^-1^. In Table 3, we list the initial components of the gas distributed throughout the tube. The ignition position was located in the front of the tube. The gas that ignites first is called the initial explosive gas, and the gas in which the subsequent (DGS) explosion occurs is called the local gas.

**Table 3:** The initial component of the gas.

Component	CH_4_	O_2_	N_2_	CO_2_	H_2_O
Mass fraction	9.5%	21%	73.7%	0	0

## 4. Simulation results and analysis

### 4.1 Local gas distribution

**Fig 11:**
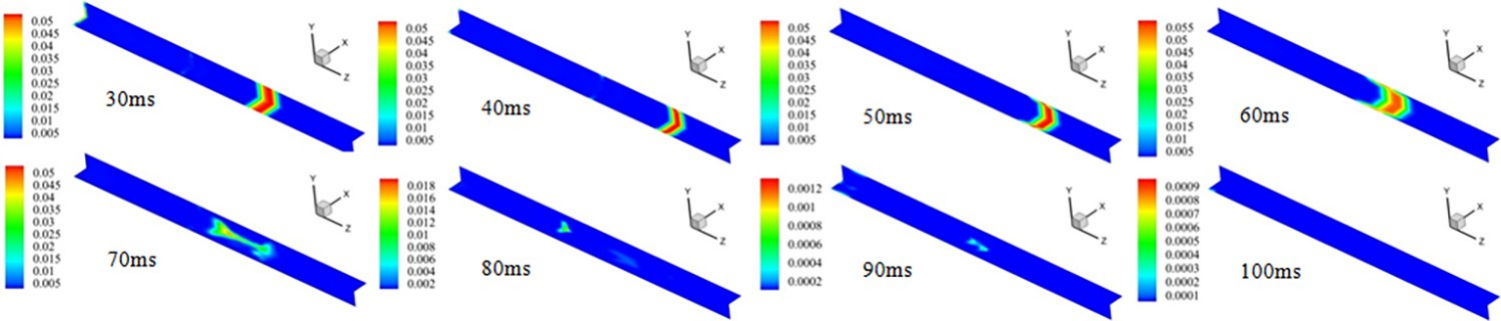
The contour of methane species at different time points.

After the initial explosive gas was ignited, shock waves and flames spread along the tube. As shown in Fig 11, the local gas is reduced in volume and moves to the tube ends at t = 30 ms, and the local gas continues to move forward at 40 ms. Compared with the previous speed, the gas at time point t = 50 ms is slower. At 60 ms, the gas content decreased, but the distribution range was the opposite. This indicates that the local gas is ignited gradually. At 70 ms, the local gas was consumed, and the shock wave product by the DGS-explosion affected the local gas distribution. The range of the local gas distribution expanded, and the distribution shape transformed from narrow to slender. This shows that the shock wave propagates forward to the end of the tube and then reflects, causing the local gas to propagate to the beginning of the tube. Subsequently, the gas was gradually consumed, and the gas in the tube almost burned out at t = 100 ms.

The distribution of the local gas was affected by the shock wave. The shock wave propagated to the end of the tube, where the local gas was ignited. The gas in front of the shock wave was compressed during propagation, generating a certain driving force. Thus, the local gas was compressed and driven forward by the original shock wave, resulting in the "gas drive" phenomenon. However, the generated shock wave propagated from the middle to both tube sides when the local gas was ignited. The shock wave propagating to the end of the tube overlapped with the original shock wave, which amplified the shock wave. The shock wave was then reflected off the wall at the end of the tube, which resulted in further amplification of the shock wave. The local gas collided with the reflected shock wave, after which the local gas migrated in the reverse direction. This changed the gas distribution and increased the risk of DGS-explosion.

### 4.2 Shock wave propagation laws

**Fig 12:**
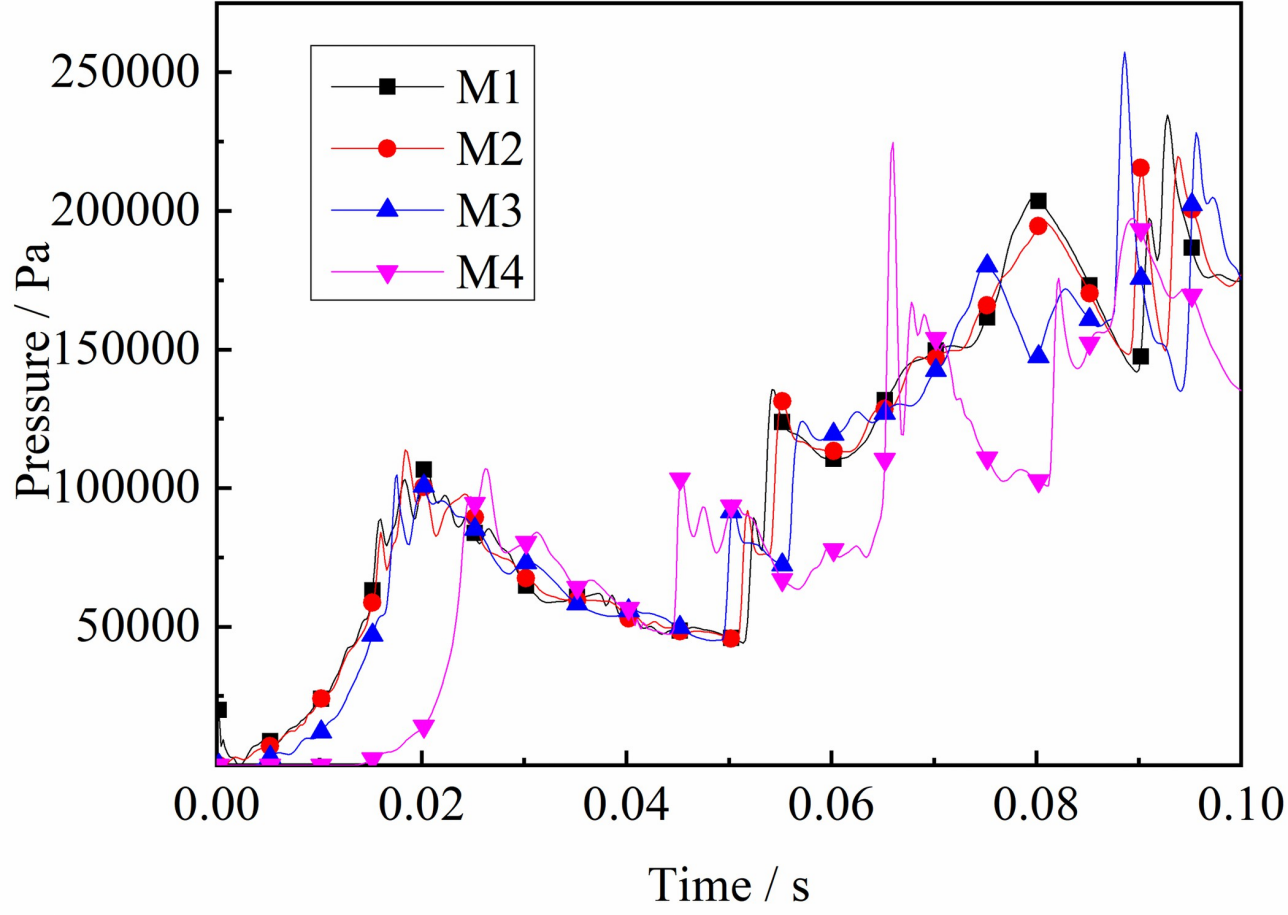
Pressure—Time curves at the monitoring point M1-M4 (gas concentrations 9.5%).

**Fig 13:**
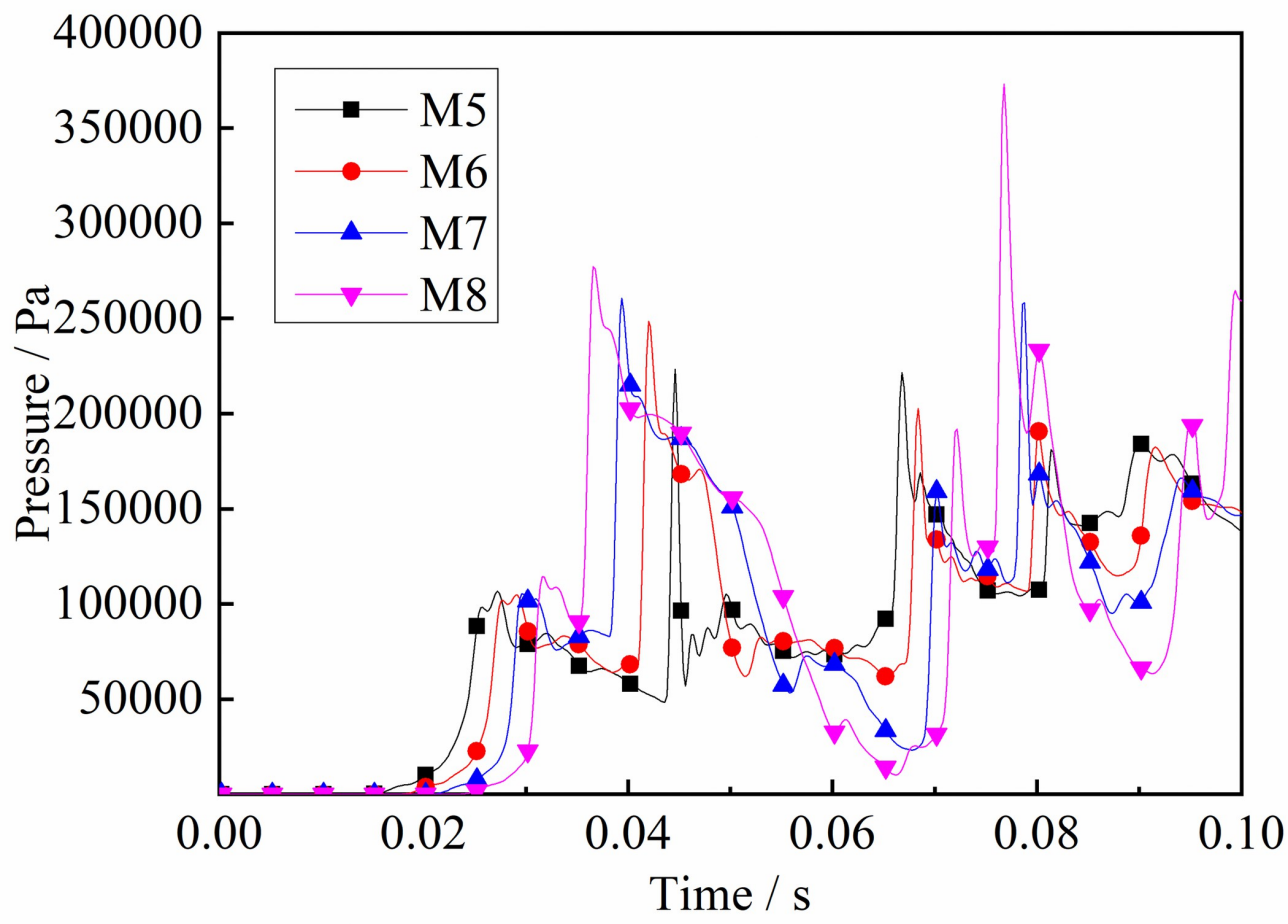
Pressure—Time curves at the monitoring point M5-M8 (gas concentrations 9.5%).

As described above, after the initial explosive gas is ignited, the shock wave travels along the tube. It can be seen from Figs 12 and 13 that the trend in pressure at both ends of the tube varies. The pressure at the monitoring point close to the ignition source began to change first. As the flame spreads forward, a DGS-explosion occurs when the local gas is ignited. The pressure variation of M5-M8 near the local gas, was different from that of the monitoring point at the front end of the tube. The pressure curve increased significantly, indicating that the pressure increased rapidly. In addition, the pressure curve at M1-M4 was weak, and the pressure increased in a relatively slow and oscillating fashion. However, the monitoring points near the local gas showed a sharp rise and fall in pressure, and the shock frequency decreased.

In addition, the maximum value of the explosion pressure in the tube is at M8. The reasons for this are as follows. First, the shock wave generated pressure at the tube ends in the initial stage. The original shock wave (in the first explosion) collides with the reflected shock wave in the tube, which reduces the pressure intensity because the waves are reversed. The flame is then stretched by the shock wave and the expended combustion area as the gas is consumed. The pressure at the monitoring point at the tube ends increases again. Finally, sufficient energy is supplied, and the peak pressure decreases. Meanwhile, as shown in Figs 12 and 13, the pressure at the monitoring point at the end of the tube is high, including the maximum pressure at M8. These results demonstrate that the shock wave has a more significant effect on the end of the tube in the DGS-explosion.

**Fig 14:**
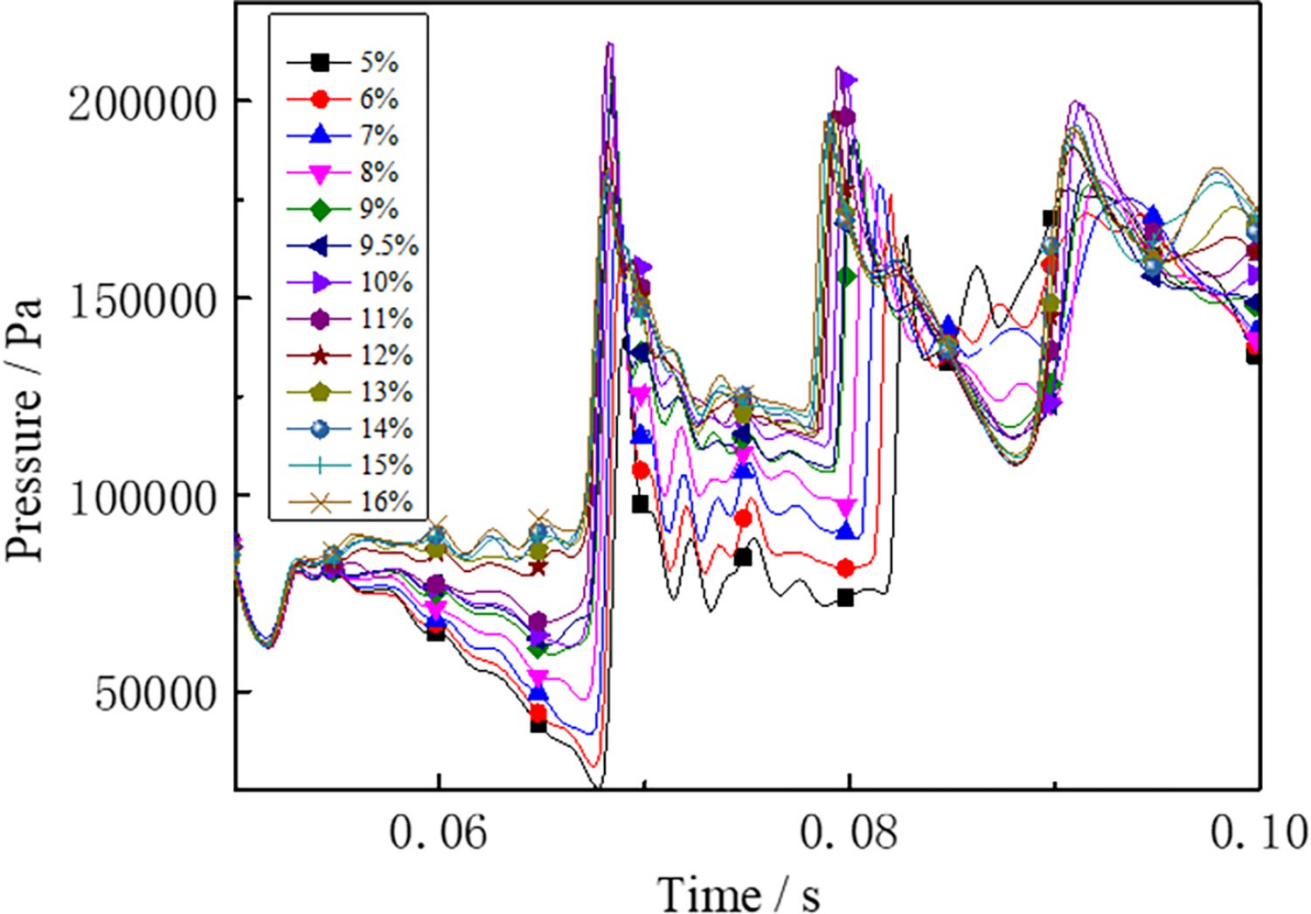
Pressure—Time curves and peak at monitoring point 6 at different gas concentrations (sequence chart).

**Fig 15:**
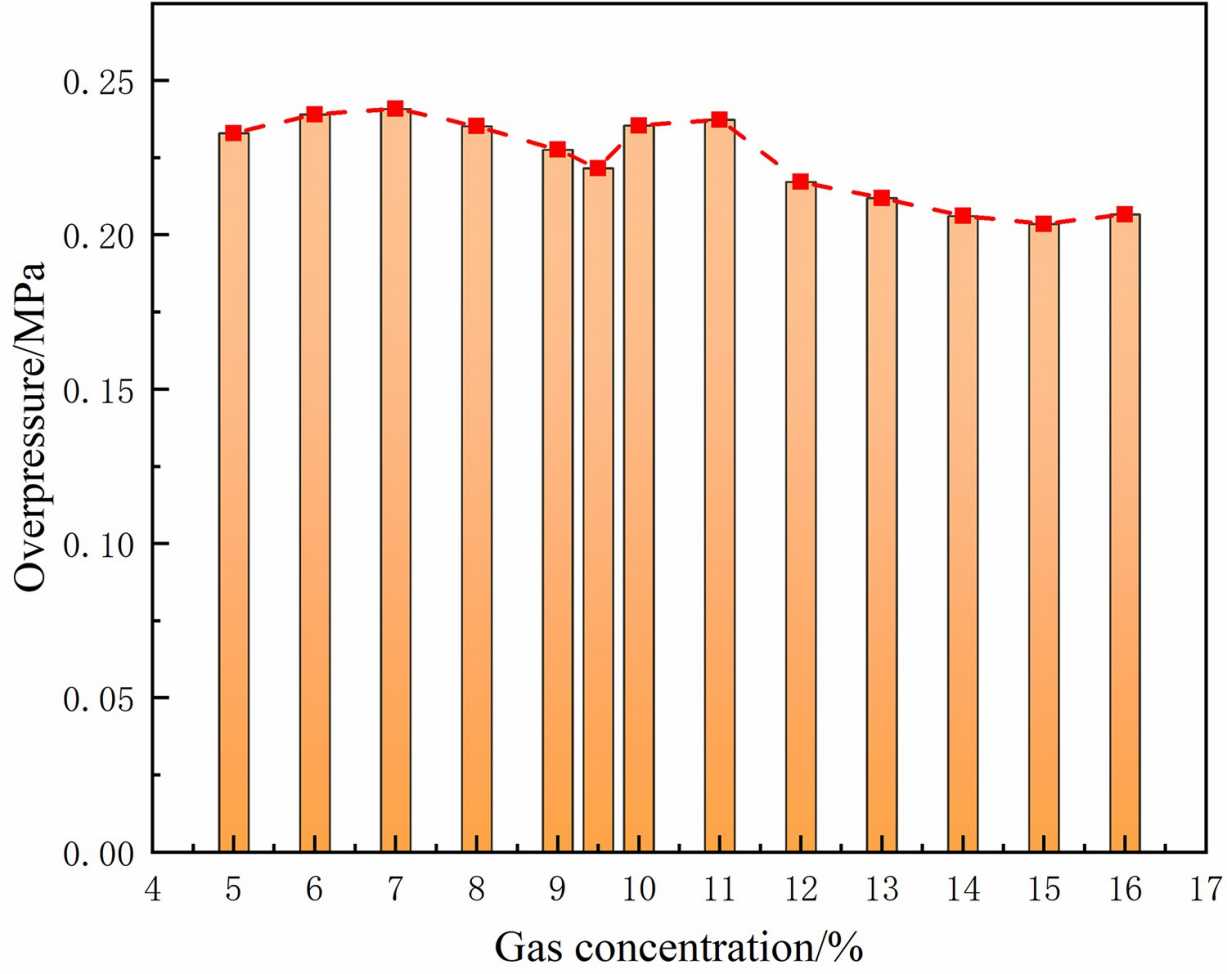
Pressure—Time curves and peak at monitoring point 6 at different gas concentrations (pressure peak).

**Fig 16:**
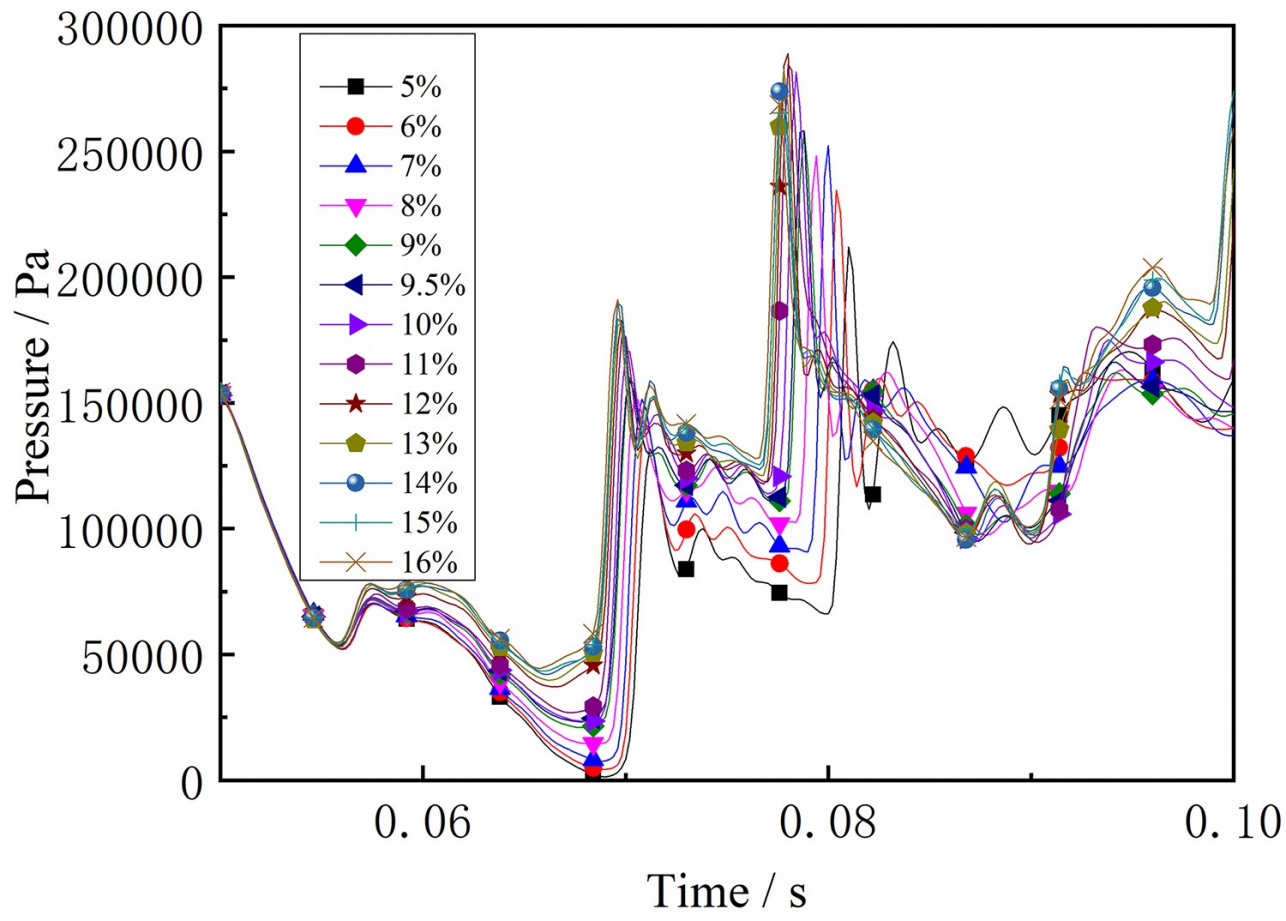
Pressure—Time curves and peak at monitoring point 7 at different gas concentrations (sequence chart).

**Fig 17:**
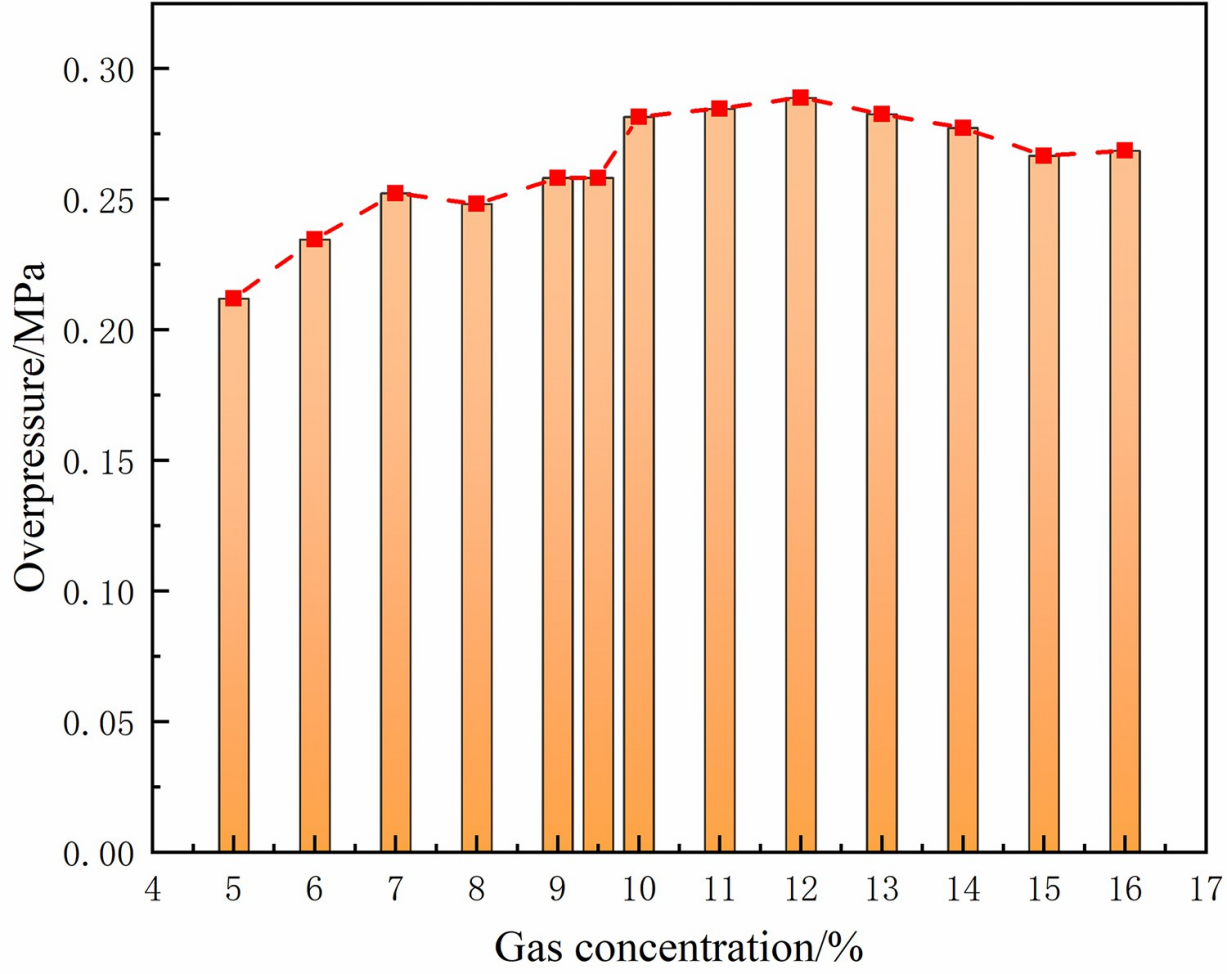
Pressure—Time curves and peak at monitoring point 7 at different gas concentrations (pressure peak).

**Fig 18:**
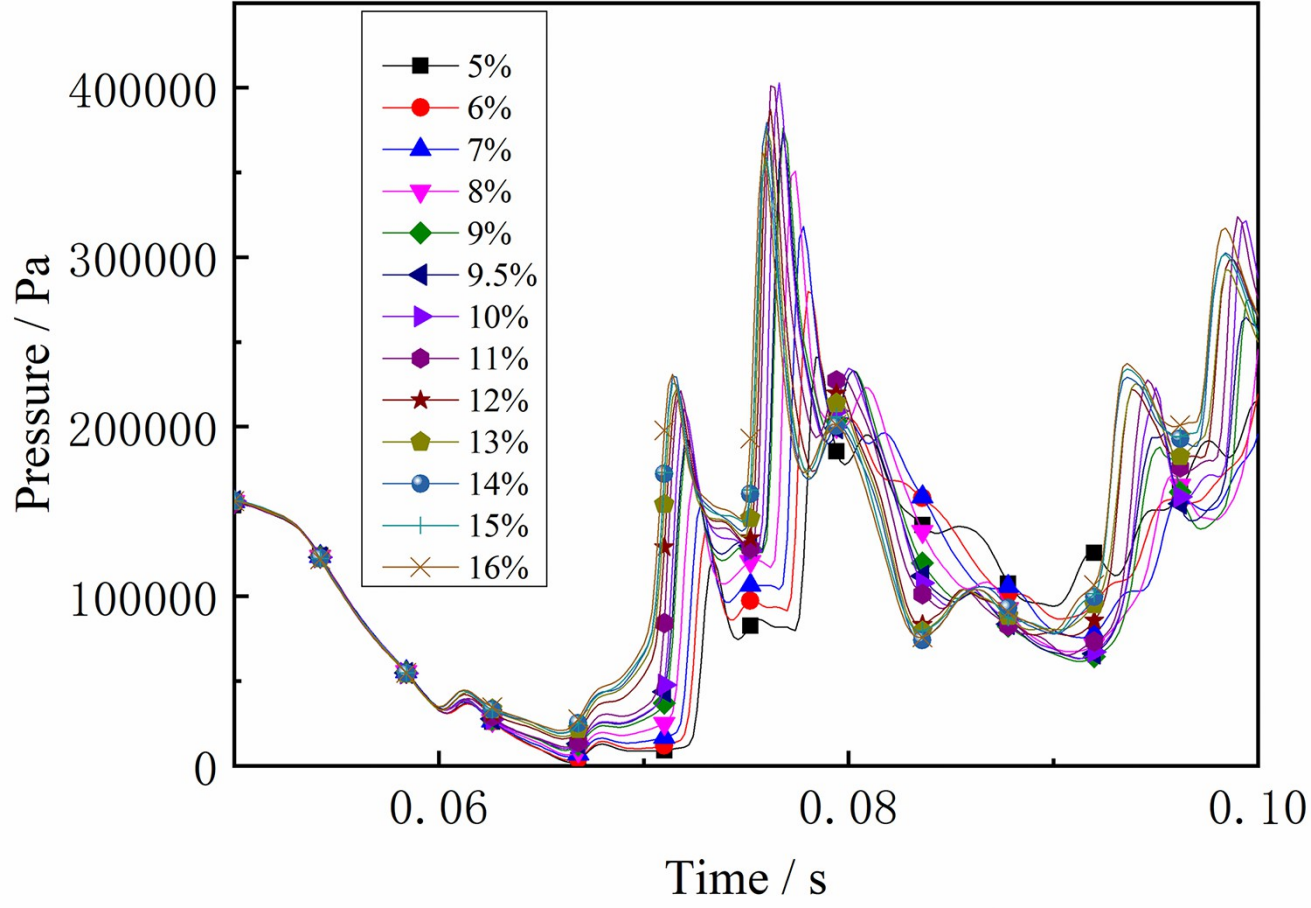
Pressure—Time curves and peak at monitoring point 8 at different gas concentrations (sequence chart).

**Fig 19:**
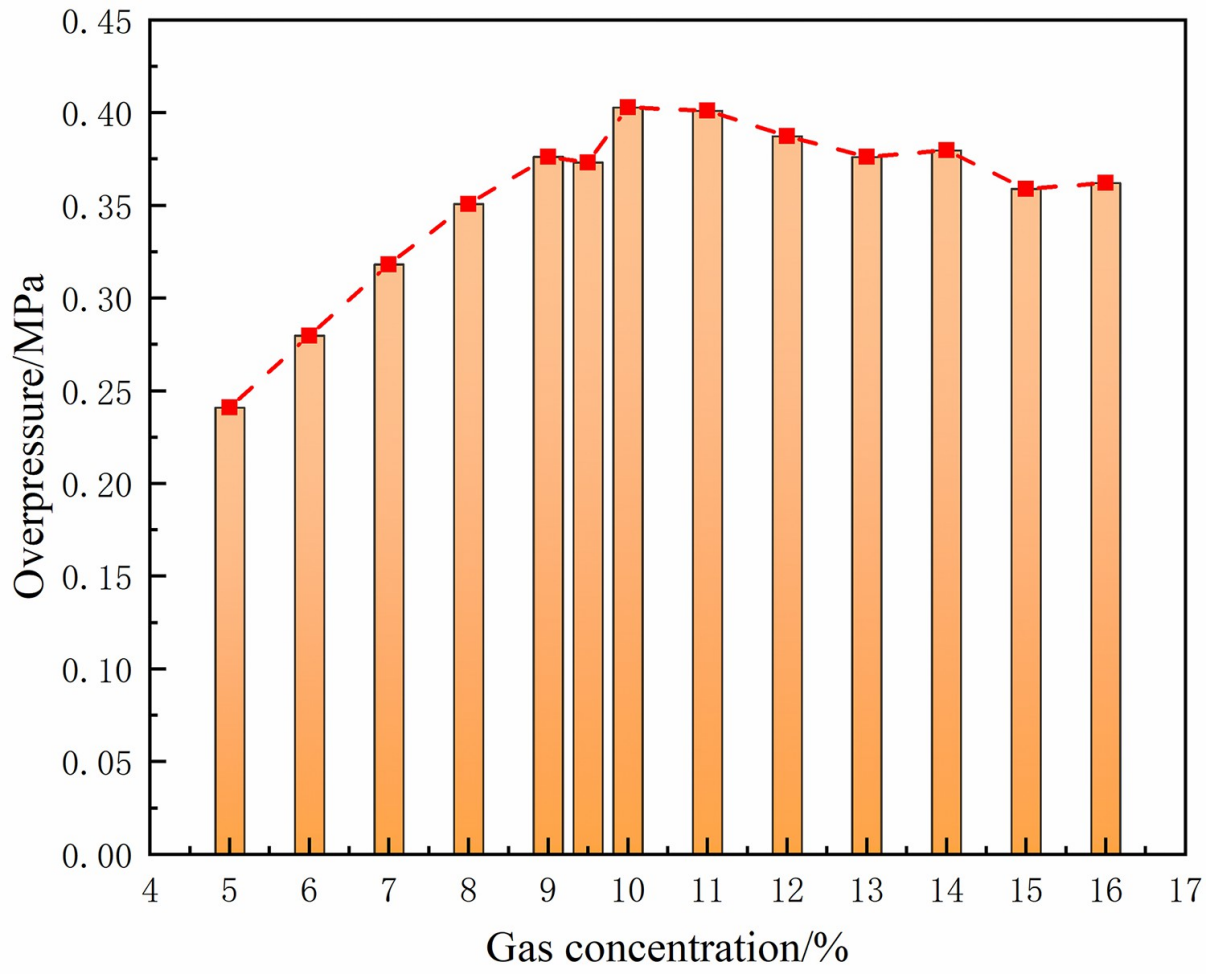
Pressure—Time curves and peak at monitoring point 8 at different gas concentrations (pressure peak).

Under the same conditions, the laws of the DGS-explosion with different gas concentrations were studied by changing the concentrations of local gas accumulation in the tube. The sequence chart and pressure peak at different monitoring points under different local gas concentrations in the tube are shown in Figs 14–19. The pressure curves at the early stage of each monitoring point in the tube coincide because the gas setting on the side at the beginning of the tube was invariable. Therefore, different pressure curves at the monitoring point were selected for the comparative analysis.

The changes in the pressure curves at the monitoring points are consistent. The changes in the pressure curve at different concentrations fluctuated constantly. The pressure shows a trend of decline-increase in the same period at each monitoring point. This is because the energy was consumed by friction loss and heat conduction as the DGS-explosion expanded outward, which led to a pressure decline. Due to the shock wave produced after the DGS-explosion, the pressure continues to increase according to the superposition principle of the shock wave. This caused an increase in pressure after the initial decline.

Moreover, the process was repeated three times as the reaction progressed, and the pressure peak decreased gradually during the rising stage. This shows that the shock wave frequency from the DGS-explosion was slower. In addition, the intensity of the pressure wave generated by the DGS-explosion gradually decreased, and the intensity of the first shock wave was the largest after the DGS-explosion. Meanwhile, the oscillation’s pressure peak and pressure curve amplitude are positively correlated with the concentration. It can be seen in the figure that after a DGS-explosion, the pressure difference is reduced by increasing the peak pressure as the concentration increases. The results show that the pressure value of the entire explosion system can be increased by changing the local gas concentration after the DGS-explosion. This indicates that the gas concentration in the DGS-explosion increases the risk of the gas explosion.

The pressure peak was reached when the DGS-explosion of gas was formed at M6, whereas the peak pressure was at M7 and M8 when the reverse shock wave. It can be seen that the rise time of the pressure at the monitoring point increases as the local gas concentration increases. The results show that the DGS-explosion will make the entire system reach a more dangerous state quickly. Moreover, the local gas concentration at the maximum pressure was 11%, 12%, and 10% at M6-M8, instead of the equivalent concentration. These results demonstrate that the DGS-explosion changes the concentration effect in the gas explosion, which underscores the need for improved safety measures to prevent situations that could lead to DGS-explosions.

### 4.3 Temperature change laws

**Fig 20:**
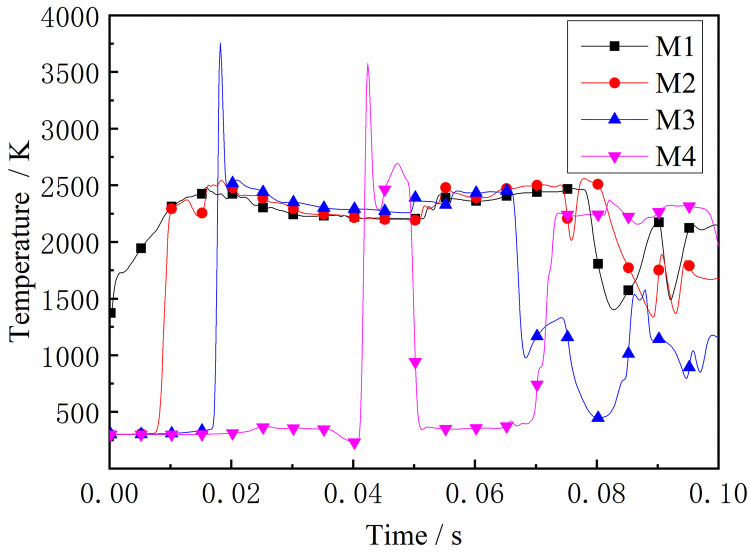
Temperature—Time curves at the monitoring point M1-M4 (gas concentration is 9.5%).

**Fig 21:**
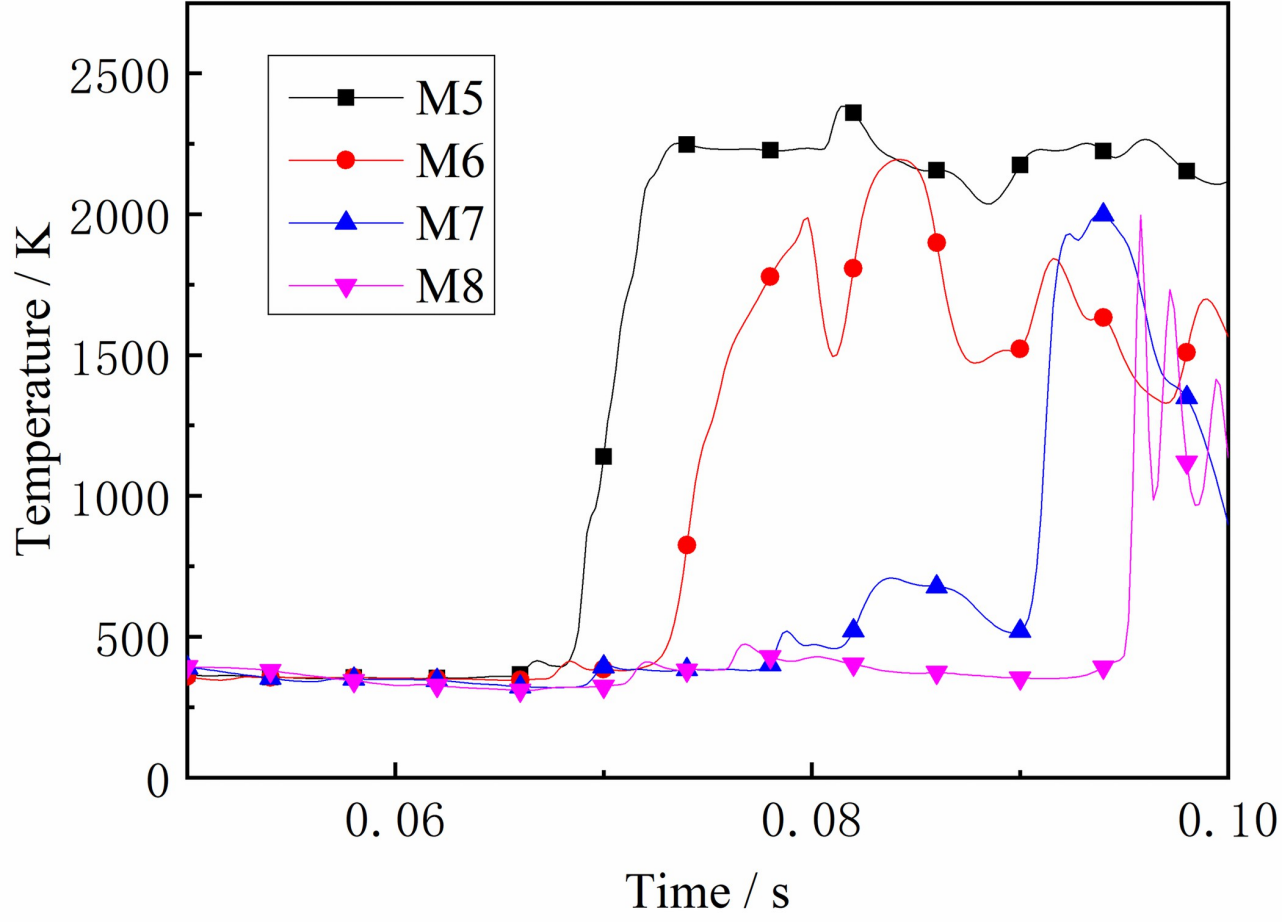
Temperature—Time curves at the monitoring point M5-M8 (gas concentration is 9.5%).

The flame front is produced in an intense chemical reaction following a shockwave. As shown in Figs 20 and 21, the flame front rapidly spread to the entire gas area as the gas was ignited around the ignition source, resulting in a rapid increase in temperature at M1. It is known that the propagation speed of the explosion pressure wave is faster than that of the flame surface, and the pressure wave pushes the high-temperature product and flame towards the end of the tube so that the high-temperature area spreads into the tube ends. Subsequently, the temperature rises. It can be seen from the figure that the temperature at M3-M6 is relatively high, indicating that the temperature in the middle of the tube has increased after the DGS-explosion. The local gas was ignited, forming a new flame front after the DGS-explosion. The newly formed flame propagated to both ends of the tube. When the flame front propagated towards the ignition source, the explosion pressure formed by the DGS-explosion overcame the original pressure. This increases the temperature at M3. Subsequently, the explosion pressure after the DGS-explosion was superimposed with the initial shock wave. The high-temperature area of the DGS-explosion began to spread towards the end of the tube, so the temperature at M4-M6 increased in the later period. The main reason for this is that the new flame front formed by the DGS-explosion affects the temperature. The DGS-explosion affects the propagation of the flame and directly affects the temperature distribution in the entire device.

**Fig 22:**
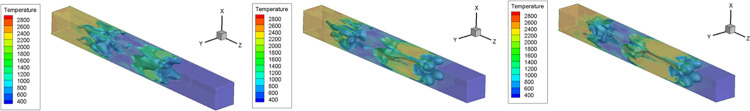
Temperature isosurface at different gas concentrations at 70 ms (5%; 9.5%; 16%).

**Fig 23:**
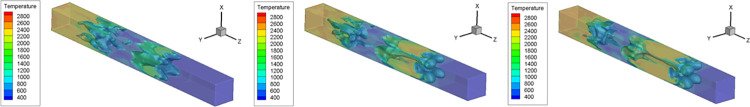
Temperature isosurface at different gas concentrations at 80 ms(5%; 9.5%; 16%).

**Fig 24:**
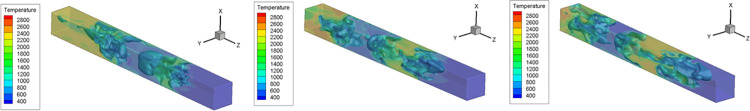
Temperature isosurface at different gas concentrations at 90 ms(5%; 9.5%; 16%).

**Fig 25:**
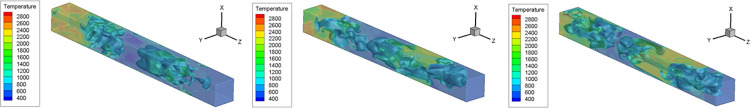
Temperature isosurface at different gas concentrations at 100 ms(5%; 9.5%; 16%).

To explore the temperature variation law of the tube with the local gas concentration, an isosurface (a plane of equal temperature) with a temperature of 1,000 K was selected for analysis. We illustrate in Figs 22–25 the isosurface of the gas explosion temperature with different local gas concentrations at different times.

**Fig 26:**
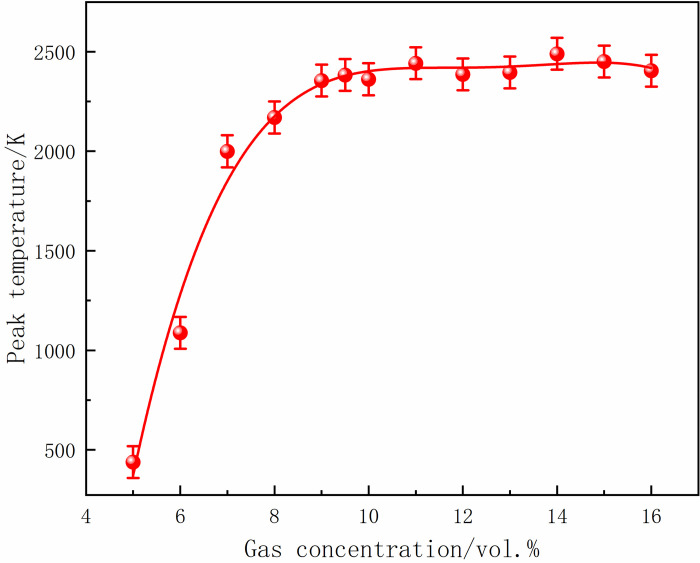
Temperature peaks at different local gas concentrations at the monitoring point 5.

**Fig 27:**
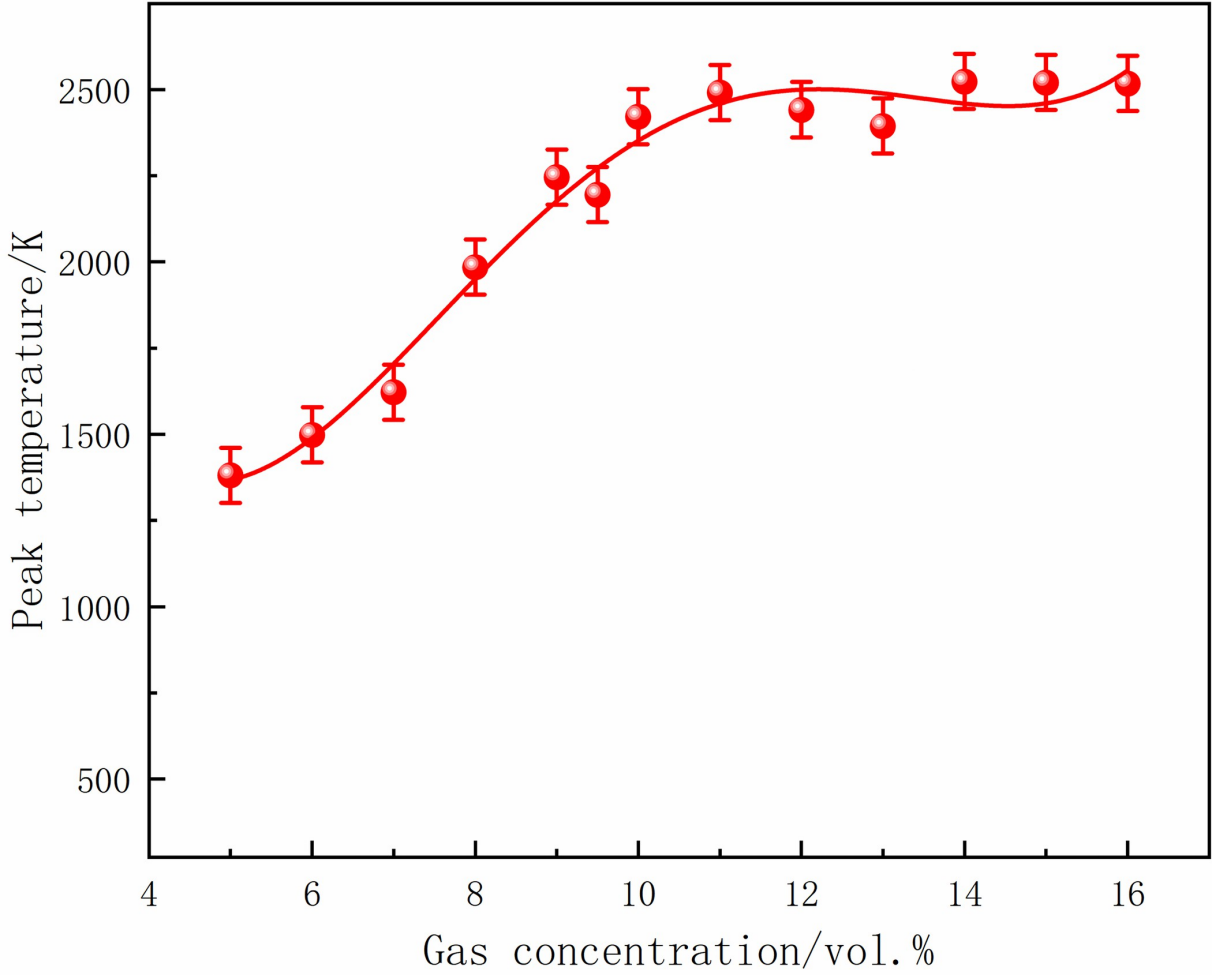
Temperature peaks at different local gas concentrations at the monitoring point 6.

**Fig 28:**
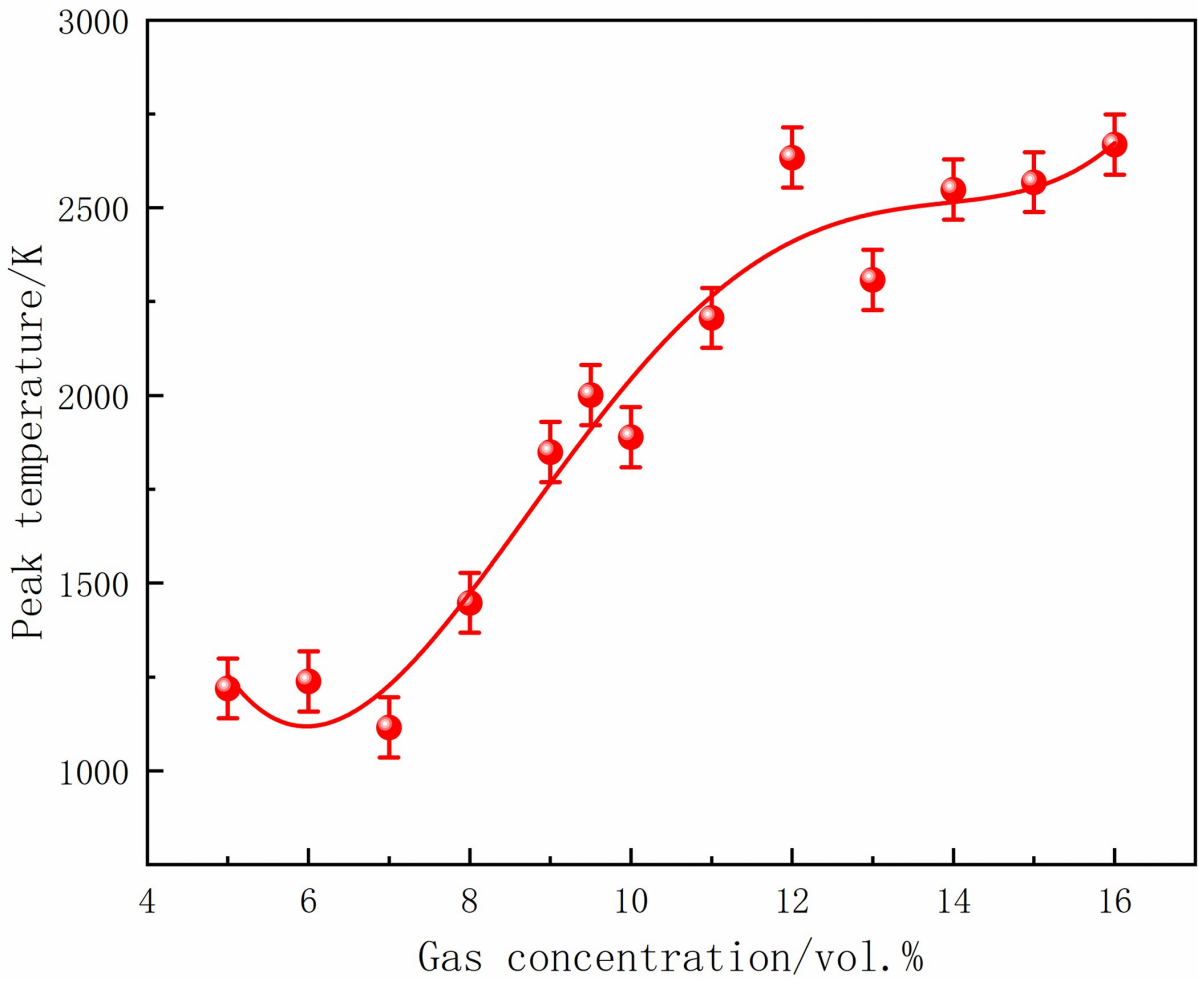
Temperature peaks at different local gas concentrations at the monitoring point 7.

**Fig 29:**
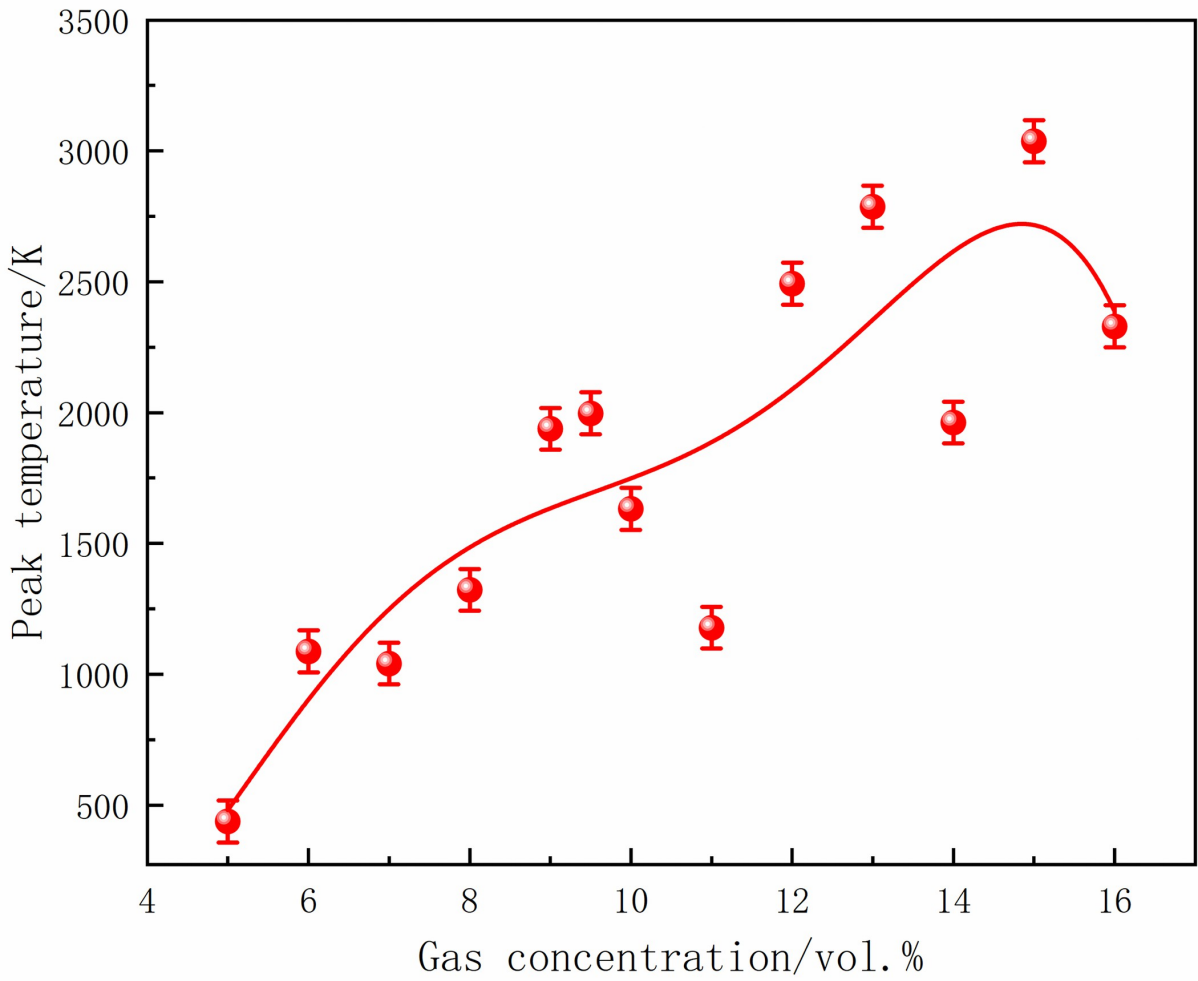
Temperature peaks at different local gas concentrations at the monitoring point 8.

At 70 ms, the gas in the center of the tube ignited and formed a flame front. The flame area was small, and the flame was concentrated when the local gas concentration was 5%. With the increase in gas concentration, the flame at the center of the ignition source was stretched, and the deformation became more complex. Moreover, the flame gradually developed, and the high-temperature area increased. After 10 ms, the flame continued to develop, and the burning area was further expanded, leading to an increase in the flame surface and folding. In addition, 16% of the gas flame region was significantly larger than 5%, and the flames at both ends were asymmetric. At 90 ms, the shock wave touched the tube end wall and propagated back. The shock wave disturbed the flame front and caused the smooth flame surface to sag inward, resulting in a wedge-shaped structure. The flame isosurface was disordered, aggravated with the increase in gas concentration, and the flame presented higher asymmetry. At 100 ms, the flame spread to both sides of the tube, further increasing the irregularity and flame distortion. The flame almost filled the tube at a concentration of 16%. The peak temperatures of M5-M8 under different local gas concentrations are shown in Figs 26–29.

The rear monitoring points did not reach the maximum temperature under the equivalent concentration. Instead, the trend was that the peak temperature gradually increased with the increase in concentration after the DGS-explosion. As can be seen from the figures above, when the local gas concentration was 5%, the peak temperature at M5 was 438 K. When the local gas concentration was 14%, the peak temperature at M5 was 2,489 K. Similar trends were observed at the M6-M8 monitoring points. It can be seen that the peak temperature of the same monitoring point generally increases with an increase in the local gas concentration. Combined with the isosurface changes in temperature at different times, it can be seen that the combustion area of the DGS-explosion increased when the local gas concentration increased, and the spread of the high-temperature flame front caused the peak temperature at the monitoring point to rise. In brief, the tube’s flame region, temperature peak, and flame irregularity increased as the gas concentration increased. The gas in the middle of the tube was ignited, forming a flame front. The flame spreads towards both sides of the tube, causing an abnormal temperature change and increasing the risk of gas explosion under the action of the explosion impact and chemical reaction formed by the flame.

## 5. Conclusions

In this study, the behavior of a local gas DGS-explosion was examined in detail, including measures of the variation in gas distribution, pressure, and temperature after the local gas DGS-explosion. The main conclusions are as follows:

1. The abnormal change in the local gas distribution was affected by the DGS-explosion. The shock wave from the DGS-explosion makes the local gas start to move towards both ends of the tube simultaneously, and the distribution accumulation changes—from the original narrow-short shape to the long-thin shape. The distribution area of the gas increased gradually. Finally, reverse migration occurs under the action of strong reflected waves until the gas is completely consumed.
2. The pressure peak is positively correlated with the gas concentration. The pressure difference was reduced by increasing the peak pressure as the concentration increased. The results show that the average pressure of the entire explosion system can be increased by changing the local gas concentration after the DGS-explosion. Moreover, it can be seen that the rise time of the pressure at the monitoring point increases as the local gas concentration increases. Finally, it is indicated that the DGS-explosion changes the concentration effect in the gas explosion, which puts forward higher requirements for disaster reduction and suppression after a gas explosion.
3. The flame region, temperature peak, and flame irregularity in the tube increased as the gas concentration increased after the DGS-explosion. With the increase in gas concentration, the flame at the center of the ignition source was stretched, and the deformation became more complex. Subsequently, the combustion area of the DGS-explosion increased when the local gas concentration increased. Universally, local gas accumulates in the heading face of underground coal mines. The outcomes of this study provide a theoretical basis for research on the influence area of the DGS-explosion, as well as for technical regulations for the prevention and control of local gas at the heading face. We anticipate that this research will contribute positively to the prevention and control of DGS-explosions.

## Supporting information

S1 Data (XLSX)

S2 Data (XLSX)

S3 Data (XLSX)

S4 Data (XLSX)

## References

[1] Zhu Y, Wang D, Shao Z, Xu C, Li M, Zhang Y. Characteristics of methane-air explosions in large-scale tunnels with different structures. Tunn undergr sp tech. 2021;109:103767. doi: 10.1016/j.tust.2020.103767

[2] Zhang Q, Zhuo C, Lang X, Li H, Qin W, Yu C. Structural Impairments of Hippocampus in Coal Mine Gas Explosion-Related Posttraumatic Stress Disorder[J]. PLoS ONE. 2014, 9(7):e102042. doi: 10.1371/journal.pone.0102042 25000505 PMC4085015

[3] Adamski R, Siuta D, Kukfisz B, Mitkowski PT, Szaferski W. Influence of process parameters in superheated steam drying on fire and explosion parameters of woody biomass. Fuel Process Technol. 2021;211:106597. doi: 10.1016/j.fuproc.2020.106597

[4] Bene De Tto AD, Garcia-Agreda A, Russo P, Sanchirico R. Combined Effect of Ignition Energy and Initial Turbulence on the Explosion Behavior of Lean Gas/Dust-Air Mixtures. Ind Eng Chem Res. 2012;51(22):7663–70. doi: 10.1021/IE201664A

[5] Lourenço A, Gominho J, Curt M, Revilla E, Villar JC, Pereira H. Steam Explosion as a Pretreatment of Cynara cardunculus Prior to Delignification. Ind Eng Chem Res. 2016. doi: 10.1021/acs.iecr.6b03854

[6] Gao K, Li S, Liu Y, Jia J, Wang X. Effect of flexible obstacles on gas explosion characteristic in underground coal mine. Process Saf Environ Prot. 2021;149:362–9. doi: 10.1016/j.psep.2020.11.004

[7] Huang C, Chen X, Liu L, Zhang H, Yuan B, Li Y. The influence of opening shape of obstacles on explosion characteristics of premixed methane-air with concentration gradients. Process Saf Environ Prot. 2021;150:305–13. doi: 10.1016/j.psep.2021.04.028

[8] Li L, Zhang Z, Liu P, Wang K, Zhang J, Li X. Experimental study of low-concentration gas explosion in large-scale pipeline. Energy Sci Eng. 2020;8(6):2129–40. doi: 10.1002/ese3.652

[9] Gao K, Liu Z, Wu C, Li J, Liu K, Liu Y, et al. Effect of low gas concentration in underground return tunnels on characteristics of gas explosions. Process Saf Environ Prot. 2021;152:679–91. doi: 10.1016/j.psep.2021.06.045

[10] Liu S, Xu J, Liu Z, Zhi L, Chen T. Research on the influence of temperature on rock strength and damage characteristics. Journal of Mining and Safety Engineering. 2013;30(4):583–8.

[11] Wang D, Zhang P, Liu S, Wei J, Sun L. Experimental study on evolutionary characteristics of pore-fissure structure in coal seam under temperature impact. Meitan Xuebao. 2018;43(12):3395–403. doi: 10.13225/j.cnki.jccs.2018.0426

[12] Ettner F, Vollmer KG, Sattelmayer T. Numerical Simulation of the Deflagration-to-Detonation Transition in Inhomogeneous Mixtures J Combust. 2014;2014:1–15. doi: 10.1155/2014/686347

[13] Cao W, Li W, Zhang Y, Zhou Z, Zhao Y, Yang Z, et al. Experimental study on the explosion behaviors of premixed syngas-air mixtures in ducts. Int J Hydrogen Energy. 2021;46(44):23053–66. doi: 10.1016/j.ijhydene.2021.04.120

[14] Chen K, Liu X, Wang L, Song D, Nie B, Yang T. Influence of sequestered supercritical CO2 treatment on the pore size distribution of coal across the rank range. Fuel. 2021;306:121708. doi: 10.1016/j.fuel.2021.121708

[15] Wang C, Wu Q, Huang B, Wang G. Numerical investigation of cavitation vortex dynamics in unsteady cavitating flow with shock wave propagation. Ocean Eng. 2018;156:424–34. doi: 10.1016/j.oceaneng.2018.03.011

[16] Kiverin AD, Yakovenko IS. Ignition and detonation onset behind incident shock wave in the shock tube. Combust Flame. 2019;204:227–36. doi: 10.1016/j.combustflame.2019.03.012

[17] Dziemińska E, Hayashi AK. Auto-ignition and DDT driven by shock wave—Boundary layer interaction in oxyhydrogen mixture. Int J Hydrogen Energy. 2013;38(10):4185–93. doi: 10.1016/j.ijhydene.2013.01.111

[18] Smirnov NN, Penyazkov OG, Sevrouk KL, Nikitin VF, Stamov LI, Tyurenkova VV. Detonation onset following shock wave focusing. Acta Astronaut. 2017;135:114–30. doi: 10.1016/j.actaastro.2016.09.014

[19] Smirnov N, Penyazkov O, Sevrouk K, Nikitin V, Stamov L, Tyurenkova V. Onset of detonation in hydrogen-air mixtures due to shock wave reflection inside a combustion chamber. Acta Astronaut. 2018;149:77–92. doi: 10.1016/j.actaastro.2018.05.024

[20] Smirnov N, Nikitin V, Stamov L, Nerchenko V, Tyrenkova V. Numerical simulations of gaseous detonation propagation using different supercomputing architechtures. Int J Comput Methods. 2017;14(04):1750038. doi: 10.1142/S0219876217500384

[21] Duan Q, Xiao H, Gong L, Li P, Zeng Q, Gao W, et al. Experimental study of shock wave propagation and its influence on the spontaneous ignition during high-pressure hydrogen release through a tube. Int J Hydrogen Energy. 2019;44(40):22598–607. doi: 10.1016/j.ijhydene.2019.06.166

[22] Xiao H, Houim RW, Oran ES. Effects of pressure waves on the stability of flames propagating in tubes. Proc Combust Inst. 2017;36(1):1577–83. doi: 10.1016/j.proci.2016.06.126

[23] Xiao H, Oran ES. Shock focusing and detonation initiation at a flame front. Combust Flame. 2019;203:397–406. doi: 10.1016/j.combustflame.2019.02.012

[24] Zhang B, Li Y, Liu H. Analysis of the ignition induced by shock wave focusing equipped with conical and hemispherical reflectors. Combust Flame. 2022;236:111763. doi: 10.1016/j.combustflame.2021.111763

[25] Wang Z, Jiao F, Cao X, Jiang K, Ma S, Yin Z. Mechanisms of vapor cloud explosion and its chain reaction induced by an explosion venting flame. Process Saf Environ Prot. 2020;141:18–27. doi: 10.1016/j.psep.2020.05.018

[26] Santner J, Goldsborough SS. Hot-spot induced mild ignition: Numerical simulation and scaling analysis. Combust Flame. 2019;209:41–62. doi: 10.1016/j.combustflame.2019.07.017

[27] Zhang L, Wang H, Chen C, Wang P, Xu L. Experimental study to assess the explosion hazard of CH4/coal dust mixtures induced by high-temperature source surface. Process Saf Environ Prot. 2021;154:60–71. doi: 10.1016/j.psep.2021.08.005

[28] Song Y, Zhang Q. Multiple explosions induced by the deposited dust layer in enclosed pipeline. J Hazard Mater. 2019;371:423–32. doi: 10.1016/j.jhazmat.2019.03.040 30875569

[29] Kindracki J, Kobiera A, Rarata G, Wolanski P. Influence of ignition position and obstacles on explosion development in methane–air mixture in closed vessels. J Loss Prev Process Ind. 2007;20(4–6):551–61. doi: 10.1016/j.jlp.2007.05.010

[30] Ajrash MJ, Zanganeh J, Moghtaderi B. Effects of ignition energy on fire and explosion characteristics of dilute hybrid fuel in ventilation air methane. J Loss Prev Process Ind. 2016;40:207–16. doi: 10.1016/j.jlp.2015.12.014

[31] Ajrash MJ, Zanganeh J, Moghtaderi B. Influences of the initial ignition energy on methane explosion in a flame deflagration tube. Energy Fuels. 2017;31(6):6422–34. 10.1021/acs.energyfuels.6b03375

[32] Askar E, Schröder V. The influence of strong ignition sources on the explosion and decomposition limits of gas es[J]. Chem Eng Trans, 2019, 77: 127–132. doi: 10.3303/CET1977022

[33] Spitzer S, Askar E, Krietsch A, Schröder V. Comparative study on standardized ignition sources used for explosion testing. J Loss Prev Process Ind. 2021;71:104516. doi: 10.1016/j.jlp.2021.104516

[34] Cao Y, Guo J, Hu K, Xie L, Li B. Effect of ignition location on external explosion in hydrogen–air explosion venting. Int J Hydrogen Energy. 2017;42(15):10547–54. doi: 10.1016/j.ijhydene.2017.01.095

[35] Addai EK, Gabel D, Kamal M, Krause U. Minimum ignition energy of hybrid mixtures of combustible dusts and gases. Process Saf Environ Prot. 2016;102:503–12. doi: 10.1016/j.psep.2016.05.005

[36] Zhao P, Tan X, Schmidt M, Wei A, Huang W, Qian X, et al. Minimum explosion concentration of coal dusts in air with small amount of CH4/H2/CO under 10-kJ ignition energy conditions. Fuel. 2020;260:116401. doi: 10.1016/j.fuel.2019.116401

[37] Zamiri A, You SJ, Chung JT. Large eddy simulation of unsteady turbulent flow structures and film-cooling effectiveness in a laidback fan-shaped hole. Aerosp Sci Technol. 2020;100. doi: 10.1016/j.ast.2020.105793

[38] Akermann K, Renze P, Dietl J, Schröder W. Large-Eddy Simulation of turbulent heat transfer in a multiple-started helically rib-roughened pipe. Int J Heat Mass Transfer. 2020;154. doi: 10.1016/j.ijheatmasstransfer.2020.119667

[39] Li S, Gao K, Liu Y, et al. Influence of the cavity structure in the excavation roadway on the gas explosion characteristics[J]. ACS omega, 2022. doi: 10.1021/acsomega.1c07027 35252714 PMC8892651

[40] Gao K, Li S, Su B, et al. Hole/pore-scale investigation of gas explosions in a coal-mine gob[J]. Process Saf Environ Prot. 2021, 156: 531–544. doi: 10.1016/j.psep.2021.10.020

